# Navigation Support during Transitions in Care for Persons with Complex Care Needs: A Systematic Review

**DOI:** 10.3390/healthcare12181814

**Published:** 2024-09-10

**Authors:** Lyndsay Howitt, Greeshma Jacob, Giulia Zucal, Judy Smith, Rhonda Crocker Ellacott, Shirlee Sharkey

**Affiliations:** 1 Registered Nurses’ Association of Ontario, 500-4211 Yonge Street, Toronto, ON M2P 2A9, Canada; gjacob@rnao.ca (G.J.); gzucal@rnao.ca (G.Z.); 2 Independent Researcher, Newmarket, ON L3Y 7T1, Canada; 3 Thunder Bay Regional Health Sciences Centre, Thunder Bay Regional Health Research Institute, 980 Oliver Road, Thunder Bay, ON P7B 6V4, Canada; rhonda.ellacott@tbh.net; 4 Independent Researcher, Stouffville, ON L4A 3T2, Canada; shirleesharkey13@gmail.com

**Keywords:** system navigation, care coordination, transitional care, healthcare delivery, continuity of patient care

## Abstract

Persons with complex care needs that arise due to chronic health conditions, serious illness, or social vulnerability are at increased risk of adverse health outcomes during transitions in care. To inform the development of a best practice guideline, a systematic review was conducted to examine the effect that navigation support has during transitions in care on quality of life, emergency department visits, follow-up visits, patient satisfaction, and readmission rates for persons with complex care needs. Eight databases were searched from 2016 to 2023. Studies were appraised using validated tools and data were extracted and presented narratively. The GRADE approach was used to assess the certainty of the evidence. Seventeen studies were included and the majority focused on transitions from hospital to home. Navigation support was provided for one month to one year following a transition. Results weakly indicate that providing navigation support during transitions in care may increase follow-up visits, reduce readmissions within 30 days, and increase patient satisfaction for persons with complex care needs. There were no important differences for quality of life and emergency department visits within 30 days of a transition. The certainty of the evidence was very low. Providing navigation support during transitions in care may improve outcomes for persons with complex needs; however, there remains uncertainty regarding the effectiveness of this intervention and more high-quality research is needed.

## 1. Introduction

A transition in care involves the transfer of a person from one setting or sector where they are receiving care or services to another setting or sector where they will receive care [1,2]. Examples include transitions from hospital to long-term care or home to hospice. During these transitions, personal information is transferred between health and social service providers, interprofessional teams, community health agencies, and various other settings [3]. Because there are often multiple health providers who work in different settings and who have various levels of accountability, a breakdown in communication and care processes can occur [2]. This can place people at an increased risk of receiving incorrect or conflicting information, unnecessary tests or treatments, medication errors, and insufficient follow-up care [2,3,4,5]. Other consequences of poor care coordination include unplanned hospital readmissions and emergency department (ED) visits and, subsequently, increased healthcare costs [2,6]. In Canada, research suggests that approximately one in eleven Canadians are readmitted to hospital within 30 days of discharge [7]. Those with complex mental or physical health conditions, language barriers, and who lack basic necessities (e.g., housing, food, and social support) are particularly vulnerable during transitions in care [2].

Patient navigation programs first emerged in North America in the 1990s as a strategy to increase cancer screening rates in underserved populations [8,9,10]. Given the growing complexity of the health system, providing navigation support has since been suggested as a strategy to promote continuity of care and help people overcome barriers as they move throughout various areas of the health system [8]. Those who provide navigation support are often called “nurse navigators” or “patient navigators” [11,12], although other regulated providers (e.g., social workers) [13] and unregulated providers (e.g., peer workers, community health workers, and transition coaches) have also fulfilled this role [8,14,15,16]. Despite various titles, the central function of this role remains the same—to reduce fragmentation of the health system by providing individualized and coordinated support to help people overcome challenges navigating the health system [8]. By regularly checking-in with a person following a transition, system navigators can determine whether a person’s needs have changed, and whether services and supports need to be reassessed [17].

In March 2021, an expert panel was convened in Ontario, Canada to update a best practice guideline on transitions in care and services [1]. The panel included people with lived experience navigating the health system as a patient or caregiver and members of the interprofessional team. While the guideline was developed to promote safe and effective transitions in care for all people navigating the health system, the panel recognized that persons with complex care needs are at particular risk of adverse events during transitions in care [2]. Thus, to inform the development of the guideline, the expert panel prioritized navigation support for persons with complex care needs as a key research area to study. Multiple scoping reviews have mapped the role and function of system navigators [8,18,19] and several systematic reviews have assessed the effectiveness of providing navigation support to specific populations, including adults undergoing cancer treatment [20,21]. However, these reviews do not focus on navigators providing support during specific transitions in care. Despite increasing research being conducted on this topic, an understanding of the effectiveness of providing navigation support to people with complex care needs during transitions in care is largely unknown.

The objective of this study is to synthesize and critically appraise the literature about the effect navigation support has during transitions in care for persons with complex care needs on the following outcomes: quality of life (QOL), ED visits within 30 days of a transition in care, follow-up visits, patient satisfaction with care or services received, and readmission rates within 30 days of a transition in care.

In this review, the term “navigation support” is defined as individualized and coordinated support provided by a system navigator to help persons overcome challenges navigating the health or social care system during their transition in care. The term “system navigator” was chosen by the panel to broadly refer to any health or social service provider, regulated or unregulated, who provides navigation support for one week or longer following a transition in care. Persons with complex care needs are defined as adults or children with chronic mental or physical health conditions, serious illness, or social vulnerability (e.g., low socioeconomic status, disability) who rely on a range of health or social care services [22,23]. These factors often lead to increased healthcare utilization, such as emergency department visits [24,25].

## 2. Methods

This systematic review was conducted and reported in accordance with the preferred reporting items for systematic reviews and meta-analyses (PRISMA) guidelines [26]. The protocol is registered in PROSPERO (CRD42021291830). The grading of recommendations assessment, development, and evaluation (GRADE) approach [27] was used to transparently and systematically rate the certainty of the evidence.

### 2.1. Role and Involvement of the Expert Panel

The expert panel determined the research question and outcomes to guide this systematic review after reviewing the research literature and findings from key informant interviews and engaging in group discussion. The expert panel included 2 people with lived experience navigating the health system as a patient or caregiver, and 18 members of the interprofessional team with expertise in clinical practice, research, education, administration, and policy. There was representation from nursing, social work, pharmacy, and medicine on the panel. Expert panel members represented a range of sectors, practice areas, and geographical areas, including urban, rural, and international settings. Both individuals with lived experience were involved in patient, family, or caregiver councils and had experience with complex transitions from hospital to home. Most clinicians on the panel reported professional experience supporting persons with complex care needs during transitions in care.

This paper reports on the methods and findings of the following question asked by the expert panel: “Should support from a system navigator be recommended or not to improve outcomes for persons encountering a transition in care?”. The question follows the PICO format (population, intervention, comparison, and outcomes) and details are outlined in Supplementary File S1. Information about how the systematic review findings were used by the expert panel to inform recommendations in the guideline can be found in the guideline’s appendices [1].

### 2.2. Search Strategy

A health science librarian conducted a comprehensive search for peer-reviewed publications from January 2016 to June 2021 using the following databases: MEDLINE, MEDLINE Epub Ahead of Print and In-Process, Embase, Emcare Nursing, Cochrane Central Register of Controlled Trials, Cochrane Database of Systematic Reviews, American Psychiatric Association PsychInfo, and Cumulative Index to Nursing and Allied Health (CINAHL). The search was limited to the last five years as per organizational protocol to develop guidelines with the most up-to-date literature. The librarian, in consultation with the authors (L.H. and G.J.) and expert panel, identified search terms including both MEDLINE MeSH headings and keywords that were relevant to transitions in care (such as “patient transfer” OR “patient discharge” OR “transitional care” OR “healthcare transition”) and system navigators (such as “patient navigation” OR “care coordinator” OR “peer support” OR “case manager”). Both search strings were combined using Boolean operators. Similar search terms were used when searching all eight databases. Expert panel members were also invited to submit articles not found by the above search strategies for eligibility consideration.

In January 2023, an update search was run to retrieve articles published between June 2021 and January 2023 to ensure the current literature was captured. The update search was conducted in CINAHL and MEDLINE only as it was not feasible for the reviewers to search all eight databases to meet the deadline for this project. There was an interval of time between completion of the initial search and commencement of the update search because the authors (L.H. and G.J.) were completing another systematic review on a different topic area during this time to inform development of another recommendation for the guideline [1]. See Supplementary File S1 for the full search strategies including the search terms used.

### 2.3. Inclusion/Exclusion Criteria and Study Selection

**Figure 1:**
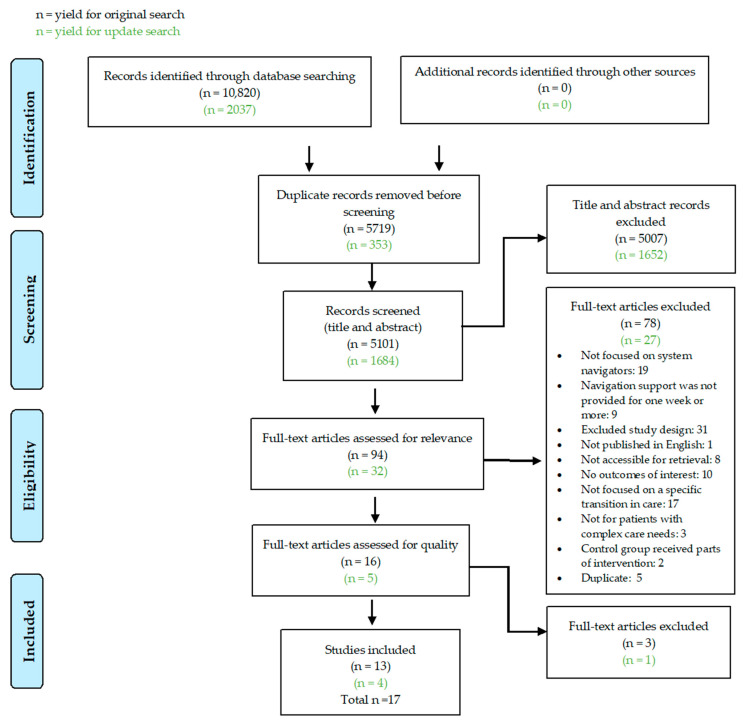
PRISMA flow diagram. Source: Adapted from [26].

The search results from all databases were combined, and duplicate studies removed. Search results were exported into DistillerSR (Evidence Partners, Ottawa, ON, Canada) for eligibility screening. Two reviewers (L.H. and G.J. for initial search, L.H. and G.Z. for update search) independently screened titles and abstracts and assessed full-text studies for eligibility using standardized screening guides. When there was a disagreement, the reviewers came to a consensus through discussion. Reasons for exclusion are outlined in Figure 1.

To be included, studies had to primarily focus on system navigators who were providing navigation support for one week or longer to an adult or child with complex care needs who was experiencing a transition between one health or social service setting to another setting where they would receive care. In this systematic review, hospitals and hospices are examples of healthcare settings. Examples of social service settings include shelters and substance use treatment centers. As this systematic review was conducted to inform the development of a guideline broadly focused on transitions in care between settings [1], inclusion criteria were intentionally kept broad and there were no restrictions placed on the types of settings persons were transitioning to or from. However, studies that did not focus on navigation support provided during a transition from one setting to another (e.g., hospital to rehabilitation facility) were excluded. The reviewers included studies where the authors of the study described participants as having (1) a complex condition or complex care needs, (2) a history of increased healthcare utilization (e.g., a high number of ED visits), or (3) a chronic condition or serious illness associated with a high risk of healthcare utilization. Healthcare utilization has been used as a proxy for complex care needs in previous research [22].

To be included, studies had to examine one or more outcomes prioritized by the expert panel, e.g., patient QOL, ED visits within 30 days of a transition in care, follow-up visits with a health or social service provider, patient satisfaction with care or services received, and readmission rates within 30 days of a transition in care [1]. The expert panel, which included people with lived experience, selected outcomes that are considered important for people experiencing a transition in care. In line with GRADE methods, the expert panel first generated a list of outcomes and then completed an online survey to rate the relative importance of each outcome. The top five outcomes were selected. The interval of 30 days was chosen by the expert panel as this timeframe is frequently studied in the literature when examining healthcare utilization [28].

Studies had to follow a quantitative or qualitative study design, be published in English, and be available for retrieval. Commentaries, editorials, narratives, case studies, pilot studies, conference abstracts, literature reviews, studies published prior to January 2016, and unpublished literature were excluded.

### 2.4. Data Extraction and Quality Appraisal

For data extraction, the included studies were divided between two reviewers (L.H. and G.J. for the initial search, L.H. and G.Z. for the update search). Each reviewer independently extracted details from their assigned studies using standardized Excel sheets (Microsoft^®^ Excel^®^ 2019 MSO (Version 2408 Build 16.0.17928.20114) 64-bit, Microsoft Corp., Redmond, WA, USA). The second reviewer independently assessed the extracted data to ensure nothing was missed. The following study details were extracted: year of publication, study design, study setting, description of the intervention and control group, patient characteristics, data collection tools, outcomes, effect size, and potential harms.

Two reviewers independently appraised the quality of all individual studies (L.H. and G.J. for initial search, L.H. and G.Z. for update search). When a discrepancy occurred, the two reviewers came to a consensus through discussion. Risk of bias was assessed for randomized controlled trials (RCT) using the Cochrane risk of bias 2.0 tool (RoB 2) [29] and for non-randomized studies using the risk of bias in non-randomized studies of interventions (ROBINS-I) tool [30]. RoB 2 assesses bias that may result during the design, conduct, and reporting of an RCT [29], while ROBINS-I is used for evaluating risk of bias in estimates of the effectiveness of an intervention from non-randomized studies [30].

The GRADE approach [27] was then used to determine the certainty of the evidence for each prioritized outcome according to each study design. L.H., G.J., and G.Z. assessed the certainty of evidence per outcome as high (⨁⨁⨁⨁), moderate (⨁⨁⨁◯), low (⨁⨁◯◯), or very low (⨁◯◯◯) depending on the extent of the following five criteria: risk of bias, inconsistency, imprecision, indirectness, and publication bias (Table 1 and Table 2) [27]. A body of evidence may begin with high certainty, but due to serious limitations in one or more of the five GRADE criteria, the evidence will be downgraded [27]. Risk of bias refers to study design or execution flaws that may bias the results, and inconsistency refers to unexplained differences in the results across studies included in the systematic review [27]. Indirectness refers to uncertainty about the applicability of the evidence to address the research question, and imprecision is the degree of uncertainty around the estimate of the effect [27]. Finally, publication bias refers to selective publication of studies based on study results [27]. Reviewers should suspect publication bias when published evidence is limited to a small number of studies all funded by industry [31]. After considering the certainty of the evidence for each individual outcome using the five criteria, L.H., G.J., and G.Z. determined the overall certainty of the evidence across outcomes.

### 2.5. Data Synthesis

Data regarding the characteristics of the included studies were summarized quantitatively using simple numerical counts. L.H., G.J., and G.Z. then developed a GRADE evidence profile to outline decisions made on the five GRADE domains. The evidence profile transparently presents decisions on how the certainty of evidence for each outcome is decided, and displays general information about the body of research evidence, including key findings and statistical results [27]. Results were summarized narratively and meta-analyses were not performed (see Supplementary File S2).

### 2.6. Ethics

There were no human participants involved in this systematic review; therefore, this study did not require informed consent or ethics approval.

## 3. Results

### 3.1. Study Selection

The search yielded 12,857 records. After duplicates were removed, the authors reviewed the titles and abstracts of 6785 records for relevance. Of those, 6659 records were eliminated, and 126 studies were reviewed in full text. After full text review, 17 studies were included (Figure 1).

### 3.2. Study Characteristics

During the systematic review, only quantitative studies were found that addressed the prioritized outcomes. Of the 17 studies included, 11 were RCTs [11,15,16,32,33,34,35,36,37,38,39], 2 were non-randomized studies with a comparison group [40,41], and 4 were single-arm studies [12,13,14,42]. Studies were carried out in the United States (n = 11) [11,12,13,15,16,33,35,36,37,38,41], Canada (n = 2) [32,42], Australia (n = 2) [14,40], the United Kingdom (n = 1) [39], and Hong Kong (n = 1) [34].

In the included studies, both regulated health and social service providers (e.g., social workers and nurses) [12,13,33,34,36,40] and non-regulated health and social service providers (e.g., transition coaches and peer workers with lived experience) [11,12,14,15,16,37,38,39,41,42] provided navigation support [1]. In nine studies, patients receiving navigation support were described as having complex conditions or complex needs [12,13,15,16,34,36,37,40,41] and many participants had a history of many ED visits or hospital readmissions. In the remaining eight studies, participants were at high risk of admission or readmission to hospital due to mental illness, chronic obstructive pulmonary disease (COPD), stroke, pneumonia, myocardial infarction, heart failure, or sickle cell disease [11,14,32,33,35,38,39,42]. In two studies, intervention was provided in communities with a high poverty rate [12,41], and, in one study, intervention was provided to children of low-income families [15]. Only one study was focused on children receiving navigation support [15]. All others focused on adults [11,12,13,14,16,32,33,34,35,36,37,38,39,40,41,42].

Twelve of the seventeen studies focused on providing navigation support during discharge from hospital to home [12,13,14,15,32,34,35,36,38,40,41,42], while the remaining studies involved transitions from hospital to home or a rehabilitation facility/skilled nursing facility [11,16,33], transitions from the ED to home [37], or discharge from a crisis resolution team to the community [39]. Crisis resolution teams provide intensive home treatment to people experiencing a mental health crisis of sufficient severity for hospital admission to be considered [39].

The way navigation support was provided by system navigators varied across the studies. System navigators primarily connected with patients in person or by phone. The duration of the intervention varied, ranging from approximately one to two months [11,14,15,16,33,36,38,40,42], three to four months [34,35,39], six months [13], and one year [12,32,37,41] following a transition in care. In four studies, system navigators connected more frequently with people experiencing a transition in the first weeks or months following the transition, with contact becoming less frequent after that [12,13,32,34]. The frequency of contact system navigators made with those they were supporting was not reported in all included studies. However, the authors of three studies noted that the frequency of contact or intervention duration were determined by the needs of the person experiencing the transition in care [13,33,40].

Four main types of support were frequently provided by the system navigators. In 13 studies, system navigators assisted with scheduling appointments, provided advice related to community resources, or made referrals to community services [11,12,13,14,16,33,34,35,37,38,40,41,42]. In 10 studies, system navigators provided individualized health education and coaching or counseling to support self-management [11,13,15,16,32,35,36,38,39,41]. In seven studies, they addressed barriers to care [11,12,35,37,38,40,41], such as transportation to and from appointments, and, in five studies, they relayed information to primary care providers to support continuity of care [11,16,32,35,40].

In the studies with a comparison group, navigation support was compared with usual care (i.e., no support from a system navigator). In the single-arm studies, results were compared pre- and post-intervention. Details from each study are outlined in Table 3.

### 3.3. Certainty of Evidence across Outcomes

The certainty of evidence for each outcome was deemed to be low or very low. The evidence was downgraded due to risk of bias, inconsistency in the results or measurement tools, and imprecision related to small sample sizes or few events. The authors also downgraded for indirectness due to differences in the intervention components and types of navigation support provided in each study. The certainty of the evidence for each outcome is further described in Section 3.4, and Supplementary File S2 provides a detailed explanation for why the evidence was downgraded. After considering the quality of evidence across all outcomes, the overall certainty of the evidence was deemed to be very low (Table 4).

### 3.4. Outcomes

Findings for each outcome are described below, listed in order of importance. These outcomes were prioritized by the expert panel, after considering outcomes important for people experiencing a transition in care.

The authors and expert panel considered the interventions examining each outcome to have enough similarities to be narratively summarized together. Each study involved a system navigator providing support during transitions in care to persons with complex care needs. A description of the interventions can be found in Table 3, but an in-depth analysis of the similarities and differences between each intervention and the impact on the outcomes of interest is beyond the scope of this manuscript.

#### 3.4.1. Patient Quality of Life

Four studies examined patient QOL [32,33,34,42]. In the studies, navigation support was provided for approximately 4 weeks [42], 12 weeks [34], 60 days [33], and 1 year [32] to persons transitioning from hospital to home. Individuals receiving navigation support had a diagnosis of COPD [32], stroke [33], severe mental illness [42], or were receiving palliative care for heart failure [34].

Overall, three RCTs showed no important differences in QOL between groups when reviewing the size of effects. When assessed using the RoB 2 tool, one RCT had high risk of bias [32] and two RCTs had some concerns [33,34]. Ng and Wong (2018) found that QOL was greater in the intervention group compared with the control group over time at 4 and 12 weeks when measured using the McGill Quality of Life Questionnaire-Hong Kong. However, when assessed using The Chronic Heart Failure Questionnaire-Chinese instrument, QOL was greater in the intervention group at 4 weeks but not at 12 weeks when compared with the control group [34]. In two other RCTs, there were no important differences in measures of QOL between the intervention and control groups [32,33]. After grading the body of evidence, the certainty of evidence for the RCTs was determined to be very low due to risk of bias, indirectness, imprecision, and inconsistency in the outcome measurement tools.

**Table 3:** Study details.

Authors, Year	Study Design	Country	No. of Participants	Intervention	Control	Outcomes of Interest
Balaban et al., 2017 [11]	RCT	USA	Intervention n = 747 Control n = 1190	For 30 days following discharge from hospital to home or a skilled nursing facility, patient navigators provided support to persons at high risk of readmission. Navigators first met with patients in hospital to assess post-discharge needs, then connected weekly by telephone. Navigators addressed barriers to medication management, arranged transportation, made connections to unspecified services, communicated with primary care providers, assisted with coordinating health insurance, and supported patient self-management.	In the control group, usual inpatient, transitional, and outpatient care was provided.	ED visits (within 30 days of a transition in care), follow-up with a health or social service provider, readmission rates (within 30 days of a transition in care).
Carter et al., 2021 [16]	RCT	USA	Intervention n = 288 Control n = 285	Community health workers supported persons at high risk for readmission transitioning from hospital to home or a short-stay rehabilitation unit for 30 days post-discharge by connecting by phone, text message, and visiting in person. They provided health coaching, arranged clinical services, and provided motivational interviewing and psychosocial support. Usual care was also provided to the intervention group.	In the control group, participants received usual post-discharge care, which included outpatient referrals.	ED visits (within 30 days of a transition in care), follow-up with a health or social service provider, readmission rates within 30 days of a transition in care)
Coller et al., 2018 [15]	RCT	USA	Intervention n = 77 Control n = 70	As a component of the Plans for Action and Care Transitions intervention, caregivers of children with medical complexity received support from a transition coach during the transition from hospital to home. Transition coaches met with patients before discharge, conducted home visits, and connected with families by phone 3 times within 30 days post-discharge. Transition coaches elicited caregiver goals, provided coaching, and reviewed medication self-management, follow-up appointments, and “red flags” that could lead to rehospitalization.	In the control group, participants received usual care which consisted of care planning, case management, and communication with community services.	Readmission rates (within 30 days of a transition in care)
Johnson et al., 2018 [39]	RCT	UK	Intervention n = 221 Control n = 220	Participants discharged from mental health crisis resolution teams received ten one-hour sessions over four months from a peer support worker. The peer support worker helped them complete a personal recovery workbook that involved creating goals, making plans to re-establish a support network, identifying early warning signs, and creating a plan to avoid relapse.	Control group participants received the recovery workbook by mail and were encouraged to complete it independently. Both intervention and control groups also received usual care.	Patient satisfaction
Kidd et al., 2016 [42]	Single-arm study	Canada	Intervention n = 31	The “welcome basket” intervention consisted of peer support workers providing weekly one-on-one support to persons with severe mental illness for four weeks following discharge from a psychiatric hospital. Peer support workers provided patients with a “welcome basket” of needed items and helped familiarize the person with community resources and supports to facilitate self-management. The vector of communication was not specified.	There was no control group. Results were compared pre- and post-intervention.	Patient QOL
LaBedz et al., 2022 [38]	RCT	USA	Intervention n = 518 Control n = 511	In the PArTNER study, hospitalized patients with pneumonia, heart failure, myocardial infarction, sickle cell disease, and chronic obstructive pulmonary disease received support from community health workers and peer coaches for 60 days post-discharge. Within hospital and after discharge, these providers addressed barriers to care, taught self-management skills, and used a personalized discharge tool to review follow-up visits, medications, tests, and lifestyle changes.	In the control group, participants received usual care from their medical team.	Follow-up with a health or social service provider
Mitchell et al., 2022 [35]	RCT	USA	Intervention n = 353 Control n = 356	The re-engineered discharge for depression intervention was delivered to participants with moderate to severe depression transitioning from hospital to home. Participants received up to 12-weeks of post-discharge telehealth support from a counselor, which included cognitive behavioral therapy, self-management support, and patient navigation. Patient navigation involved arranging transportation, scheduling appointments, and relaying information to primary care providers.	In the control group, participants received a post-discharge telephone call from a discharge educator, who reviewed medication, confirmed follow-up appointments, and provided education about symptoms and the plan of care.	ED visits (within 30 days of a transition in care), readmission rates (within 30 days of a transition in care)
Ng & Wong, 2018 [34]	RCT	Hong Kong, China	Intervention n = 41 Control n = 43	In the home-based palliative heart failure program, persons with end-stage heart failure received home visits and phone calls from a palliative nurse case manager over a 12-week period after transitioning from hospital to home, in addition to usual care. The nurse case manager assessed symptoms, provided spiritual and social support, discussed goals and treatment preferences, and made referrals to other providers and services when necessary.	The control group participants received usual care and two social calls. Usual care involved a pre-discharge palliative care referral, standard discharge planning, and an outpatient palliative care clinic appointment.	Patient QOL, patient satisfaction
Pang et al., 2023 [40]	Cohort study	Australia	Intervention n = 63 Control n = 262	Persons at high risk of hospital readmission received targeted care coordination, which involved weekly phone calls for 30 days post-discharge by a registered nurse, who coordinated and addressed barriers to attending follow-up appointments. To promote continuity, the nurse also relayed information to general practitioners.	In the comparison group, usual care was provided, which included follow-up appointments with the treating ward team. Persons booked their own appointments or had appointment instructions mailed to them.	Follow-up with a health or social service provider
Reeves et al., 2019 [33]	3-group RCT *	USA	Intervention n = 88 Control n = 87	During the Michigan Stroke Transition Trial, stroke patients transitioning from hospital to home or a rehabilitation facility received case management support from a social worker which included a biopsychosocial assessment, development of a care plan based on patient goals, assistance arranging appointments and community service referrals, and practical and emotional support. Support was provided through home visits and phone calls for approximately 60 days after returning home from hospital.	In the control group, usual care consisted of providing standard post-discharge instructions about medications, follow-up appointments, and referrals.	Patient QOL
Rose et al., 2018 [32]	RCT	Canada	Intervention n = 236 Control n = 234	In addition to usual care, patients in the intervention group with chronic obstructive pulmonary disease received support from a case manager for one year after discharge home from hospital. The case manager provided education and individualized action plans, telephone consultations, and motivational interviewing focused on self-management. They also relayed information to family doctors and hospital specialists.	Usual care consisted of three monthly outpatient clinic visits, an eight-week rehabilitation program, and an individualized action plan that included education materials.	Patient QOL, patient satisfaction
Samuels et al., 2021 [12]	Single-arm study **	USA	Intervention n = 49 Control n = 51	Patient navigators provided 12 months of support to frequent ED users after discharge from the ED. Patient navigators scheduled and reminded persons about medical appointments, and accompanied patients to appointments if necessary. They routinely contacted patients by phone to assess needs and address barriers to care, and made referrals as required.	Standard care was provided to the control group.	Patient satisfaction
Scanlan et al., 2017 [14]	Single-arm study	Australia	Intervention n = 64	Following discharge from an inpatient psychiatric unit, patients received six to eight weeks of support from a peer worker who had their own lived experience of mental illness and recovery. Supports were tailored to each person but were mostly focused on providing emotional and practical support and connections to community resources.	There was no control group.	Patient satisfaction
Seaberg et al., 2017 [37]	RCT	USA	Intervention n = 148 Control n = 134	Patient navigators provided support to persons who had five or more ED visits within 12 months. During the initial ED visit, any subsequent visits, and by phone at 2 weeks and 12 months, navigators reviewed the patients’ diagnoses and care plans, arranged follow-up appointments and transportation, and linked patients with community resources.	Standard care was provided to the control group.	Follow-up with a health or social service provider, patient satisfaction
Taylor et al., 2022 [36]	RCT	USA	Intervention n = 349 Control n = 342	In the sepsis transition and recovery program, persons with sepsis at high risk of readmission received usual care plus telephone support from a sepsis nurse navigator for 30 days post-discharge. The navigator promoted care planning and self-management, coordinated follow-up care, assessed and treated health concerns, and escalated care when needed.	The control group received usual care at discharge which often included patient education, follow-up instructions at discharge, and referrals for home care services.	ED visits (within 30 days of a transition in care), follow-up with a health or social service provider, readmission rates (within 30 days of a transition in care)
Thompson et al., 2018 [41]	Cohort study	USA	Intervention n = 159 Control n = 280	Community navigators supported super utilizers (people with 11 or more hospital encounters in one year following an ED encounter or hospital admission). For one year, navigators connected clients with community resources, addressed barriers to care, tailored health information to client needs, and encouraged clients to adopt healthy behaviors.	The comparison group consisted of data from super utilizers from neighboring zip codes who did not receive the intervention.	Readmission rates (within 30 days of a transition in care)
Xiang et al., 2019 [13]	Single-arm study	USA	Intervention n = 586	The bridge model for super utilizers is a social work-based transitional care intervention, implemented to address the medical and social needs of super utilizers (persons with ≥5 hospital admissions over 12 months), over a 6–12-month period. The social worker assessed needs, coordinated care, provided counseling, and organized services and referrals.	There was no control group, and results were compared pre- and post-intervention.	Readmission rates (within 30 days of a transition in care)

Abbreviations: randomized controlled trial (RCT); quality of life (QOL); emergency department (ED). * This was a 3-group RCT, where the 3rd group received intervention plus access to a patient-oriented website with stroke information. Data from the 3rd group were not focused on for this systematic review. ** This was a single-arm evaluation of patient satisfaction in the intervention group of a RCT.

**Table 4:** Certainty of evidence for prioritized outcomes using GRADE criteria.

	Patient Quality of Life		Emergency Department Visits (Within 30 Days of a Transition in Care)	Follow-Up Visit with a Health or Social Service Provider		Patient Satisfaction		Readmission Rates (Within 30 Days of a Transition in Care)		
Study Design	**RCT**	**Single-Arm Study**	**RCT**	**RCT**	**Cohort Study**	**RCT**	**Single-Arm Study**	**RCT**	**Cohort Study**	**Single-Arm Study**
No. of studies	3	1	4	5	1	4	2	5	1	1
Risk of bias	−1	−1.5	−1	−2	−1	−1.5	−2	−1.5	−1.5	−1.5
Inconsistency	−0.5	0	0	−1	0	−2	−0.5	−1.5	0	0
Indirectness	−0.5	−0.5	−0.5	−0.5	−0.5	−0.5	−0.5	−0.5	−0.5	−0.5
Imprecision	−0.5	−2	0	0	−1.5	0	−2	0	−0.5	1
Publication bias	Undetected	Undetected	Undetected	Undetected	Undetected	Undetected	Undetected	Undetected	Undetected	Undetected
Certainty of evidence	Very low ⨁◯◯◯	Very low ⨁◯◯◯	Low ⨁⨁◯◯	Very low ⨁◯◯◯	Very low ⨁◯◯◯	Very low ⨁◯◯◯	Very low ⨁◯◯◯	Very low ⨁◯◯◯	Very low ⨁◯◯◯	Very low ⨁◯◯◯
Overall certainty of evidence across outcomes: Very low ⨁◯◯◯										

A value of zero indicates no downgrading for this criterion. Negative values indicate the evidence was downgraded by 0.5, 1, or 2 levels for this criterion. Abbreviations: GRADE—grading of recommendations assessment, development, and evaluation; RCT—randomized controlled trial.

One single-arm study by Kidd et al. (2016) reported an increase in QOL when measured pre- and post-intervention, such that a large effect size was observed in the living situation subscale and a low-medium effect size was observed in the social relationship subscale when measured using the Satisfaction With Life Scale. No differences were seen in the remaining subscales [42]. The certainty of evidence for this non-randomized study was very low due to risk of bias, indirectness, and imprecision due to a small sample size.

#### 3.4.2. Emergency Department Visits (Within 30 Days of a Transition in Care)

Four RCTs studied ED visits within 30 days of a transition in care [11,16,35,36]. In the included studies, navigation support was provided for 30 days [11,16,36] and 12 weeks [35] to persons with moderate to severe depression [35], sepsis [36], and persons at high risk of hospital readmission [11,16]. Support was provided following a transition from hospital to home or a rehabilitation facility/skilled nursing facility [11,16,35,36].

Mitchell et al. [35] found no important differences in ED visits within 30 days of a transition in care in the intervention group compared with the control group (10% vs. 9%, IRR 1.05; 95% CI, 0.64–1.72), neither did Taylor et al. [36] (OR 1.12; 95% CI, 0.71–1.78). In Carter et al.’s RCT [16], there was a trend towards a reduction in ED visits within 30 days of discharge in the intervention group compared with the control group, but this was not statistically significant (11.2% vs. 16.8%; OR, 0.62; 95% CI, 0.38–1.02). Finally, Balaban et al. [11] found a non-statistically significant decrease in ED visits within 30 days of a transition in care in the intervention group compared with the control group, but only among persons 60 years and older. When results across studies were reviewed together, there were no important differences in ED visits between participants who received navigation support during transitions in care and those who did not. Three of the RCTs had some concerns about risk of bias [11,16,36] and one had low risk of bias [35]. After grading the evidence, the certainty was determined to be low due to risk of bias in the included studies and indirectness.

#### 3.4.3. Follow-Up Visit with a Health or Social Service Provider

Six studies explored the effect that navigation support has during transitions in care on outpatient follow-up visits with a health or social service provider [11,16,36,37,38,40]. Navigation support was provided for 30 days [11,16,36,40], 60 days [38], and 1 year [37] following a transition from hospital to home or a rehabilitation facility/skilled nursing facility [11,16,36,37,38,40]. Participants receiving navigation support were either at high risk of being readmitted to hospital or returning to the ED [11,16,37,40], or had a diagnosis of sepsis [36], heart failure, pneumonia, COPD, myocardial infarction, or sickle cell disease [38].

One RCT with some concerns related to risk of bias reported no important difference in outpatient visits within 14 days of hospital discharge between the intervention and control groups (OR 1.0; 95% CI, 0.78–1.3) [38]. However, four RCTs, [three with some concerns related to risk of bias [11,16,36] and one with high risk of bias [37] found an improvement or trend towards an improvement for this outcome [11,16,36,37]. Carter et al. [16] reported fewer missed outpatient appointments within 30 days of discharge from hospital in the intervention group compared with the control group (OR 0.56; 95% CI, 0.38–0.81). Taylor et al. [36] noted more outpatient follow-up visits within 10 days of hospital discharge in the intervention group in contrast with the control group (39% vs. 31%). Seaberg et al. [37] reported increased primary care visits over a 12-month follow-up period in the intervention group (6.42 visits/patient) compared with the control group (4.07 visits/patient). In the RCT by Balaban et al. [11], outpatient visits in the first 30 days post-discharge were higher in the intervention group among adults 60 years and older. After grading the body of evidence, the certainty of the evidence for RCTs was very low due to risk of bias, indirectness, and inconsistency in the effects and tools used for outcome measurement.

In one cohort study, more patients in the intervention group had a follow-up medical appointment scheduled within 90 days post-discharge compared with those in the comparison group (66.7% vs. 36.6%) [40]. The certainty of the evidence for this non-randomized study was very low due to risk of bias, indirectness, and imprecision related to a small number of events.

#### 3.4.4. Patient Satisfaction

Six studies (four RCTs [32,34,37,39] and two single-arm studies [12,14]) examined patient satisfaction. Navigation support was provided for 6 to 8 weeks [14], 12 weeks [34], 4 months [39], and 1 year [12,32,37] following transition from the ED or hospital to home [12,14,32,34,37] or from a mental health crisis resolution team to the community [39]. Individuals receiving navigation support had a diagnosis of mental illness [14,39], COPD [32], were receiving palliative care for heart failure [34], or were frequent users of the ED [12,37].

There was inconsistency in the results among the four RCTs. Two studies with some concerns related to risk of bias reported an increase in satisfaction in the intervention group compared with the control group [34,39], while two studies with high risk of bias showed no important between-group differences [32,37]. The certainty of the evidence for RCTs was very low due to risk of bias, indirectness, and inconsistency. Both single-arm studies reported patient satisfaction after participants received navigation support [12,14], but the certainty of the evidence was very low due to risk of bias, indirectness, inconsistency in the tools used to measure outcomes, and imprecision related to small sample sizes.

#### 3.4.5. Readmission Rates (Within 30 Days of a Transition in Care)

Seven studies reported on readmissions to hospital within 30 days of a transition in care [11,13,15,16,35,36,41]. Navigation support was provided to persons who were frequent users of the health system, persons deemed to be at high risk of hospital readmission [11,13,16,41], or persons with a diagnosis of sepsis [36], moderate to severe depression [35], or medical complexity [15]. Individuals receiving navigation support were transitioning from hospital to home or a rehabilitation facility/skilled nursing facility [11,13,15,16,35,36,41] and received navigation support for approximately 1 month [11,15,16,36], 3 months [35], six months [13] and one year [41].

Three RCTs that had some concerns related to risk of bias reported a reduction or a trend towards a reduction in 30-day hospital readmissions in the intervention group compared with the control group, e.g., Coller et al. [15] (adjusted IRR: 0.37; 95% CI, 0.14–0.98), Carter et al. [16] (OR 0.44; 95% CI, 0.28–0.90), and Taylor et al. [36] [71 readmissions (20.3%) vs. 84 (24.6%), OR 0.78; 95% CI, 0.55–1.12]. One RCT by Mitchell et al. [35] that had low risk of bias reported no important differences in readmissions between the intervention and control groups at 30 days (10% vs. 9%, IRR 0.92; 95% CI, 0.56–1.52), while another RCT by Balaban et al. [11] with some concerns related to risk of bias reported increased readmissions 30-days post-discharge in the intervention group compared with the control group (0.312 events per person vs. 0.158 events per person, *p* = 0.002), but only among adults less than 60 years. The certainty of the evidence for RCTs was very low due to risk of bias, indirectness, and inconsistency.

In one cohort study, the intervention group had an 18% greater reduction in 30-day readmissions compared with the comparison group, but this difference was not statistically significant (95% CI, −44% to 22%) [41]. In Xiang et al. [13]’s single-arm study, there was a 17% reduction in the 30-day readmission rate 1 month after intervention. The certainty of the evidence for the non-randomized studies was very low due to risk of bias, indirectness, and imprecision.

#### 3.4.6. Intervention Duration and Prioritized Outcomes

Given the heterogeneity observed in the interventions reviewed, the authors attempted to explore how intervention differences might influence outcomes. The authors explored the outcomes of patient QOL and patient satisfaction with consideration to how long the intervention was delivered (see Supplementary File S3). Within the four studies [32,33,34,42] that examined patient QOL, there was no indication of a potential relationship. For the outcome of patient satisfaction, in two out of three studies where the intervention was delivered for one year, no important differences were noted in satisfaction [32,37]. However, important differences in satisfaction were noted in the studies where the intervention was delivered for four months or less [14,34,39]. Given that only six studies examined patient satisfaction, and the interventions varied in other ways, it is unclear whether a potential relationship could exist. The relationship between intervention duration and 30-day ED rates, readmission rates, and follow-up visits was not considered since the timeframe of these outcomes likely precluded impact by intervention duration.

## 4. Discussion

The purpose of this systematic review was to synthesize the evidence about the effectiveness of navigation support during transitions in care for persons with complex care needs on the outcomes of patient QOL, ED visits within 30 days of a transition in care, follow-up visits with a health or social service provider, patient satisfaction with care or services received, and readmission rates within 30 days of a transition in care. Seventeen studies were identified, but none examined all outcomes of interest in one study. The results of this systematic review suggest that providing navigation support during transitions in care may increase follow-up visits with a health or social service provider [11,16,36,37,40], reduce hospital readmissions within 30 days of a transition in care [13,15,16,36], and increase patient satisfaction [12,14,34,39] for persons with complex care needs, but the evidence is very uncertain. Certainty in these findings was limited as the quality of the evidence was very low due to risk of bias, indirectness, inconsistency in results and measurement tools, and imprecision related to small sample sizes and number of events. There were inconsistencies in the results, with some studies showing no important differences in follow-up visits [38], patient satisfaction [32,37], or readmissions within 30 days of a transition in care [35]. Moreover, after reviewing the size of effects across studies, there were no important differences found regarding the effect of system navigators on QOL and ED visits within 30 days of a transition in care.

It is unclear why some studies demonstrated a beneficial effect when receiving navigation support, while others reported no important differences. Heterogeneity in the interventions between studies, and the differences in the individual components of the interventions within studies makes comparing the results challenging. Not only did the type of support provided by system navigators vary across studies, but the healthcare contexts that support was provided in and the providers who delivered the support (i.e., regulated and unregulated health or social service providers) differed between studies. In addition, those receiving navigation support had a variety of complex needs, and received support from system navigators for different lengths of time. Despite differences across studies, there were some similarities in the components of navigation support, including scheduling appointments, providing guidance about community resources or making referrals to community services [11,12,13,14,16,33,34,35,37,38,40,41,42], providing health education or coaching to support self-management [11,13,15,16,32,35,36,38,39,41], addressing barriers to care [11,12,35,37,38,40,41], and relaying information to primary care providers to support continuity of care [11,16,32,35,40]. In Couturier et al. [43]’s scoping review about care coordination for patients with complex needs in primary care, authors rarely provided a rationale for why specific care coordination activities were performed in the included studies. The same is true for this review; the rationale for the types of activities performed by system navigators were seldom described. Couturier et al. [43] concluded that, in general, care coordination involves arranging services, developing a relationship with the client, and advocating on the client’s behalf within a complex system; the same actions were routinely performed by system navigators during transitions in care in this review.

Within the body of the literature, some concerns exist as to whether system navigators serve as a “band-aid” solution for fragmented health systems, and whether they divert attention and funding from system-level changes that are needed to make the health system easier for all to navigate [8,44]. Developing new models of care has been proposed as one system-level strategy to reduce fragmentation of the health system [44]. Many current models of care are disease-focused and target specific body systems, yet often people have complex care needs that cannot be addressed in isolation [44]. As populations age and more individuals develop age-related complex care needs, developing new holistic and integrated models of care may help address health and social care needs and alleviate the necessity of system navigators [44].

While a debate may remain as to whether navigation support simply provides a temporary solution for a fragmented health system, the views of patients and caregivers receiving navigation support must also be acknowledged. In the studies included in this review, patients and caregivers who received navigation support during transitions in care noted that system navigators helped bridge inpatient and outpatient services [42], helped make connections to needed community-based resources [14], and provided emotional and practical support (e.g., assistance with transportation) [14]. In Samuels et al. [12]’s study, participants viewed system navigators as advocates, and felt relieved upon finally receiving the individualized support and continuity of care they required. It is clear that support is required in some manner to reduce the fragmentation of care and help persons with complex needs overcome barriers when navigating the health system [8]. The development of navigation models highlights the unmet needs of those most vulnerable and the need for support during transitions in care [8].

Several scoping reviews have explored what processes facilitate the implementation of system navigation programs [45,46]. Providing training and role clarity for navigators, promoting effective communication between navigators and other health providers, and securing sufficient financial resources and funding are factors that contribute to implementing and sustaining navigation programs [45,46]. In addition, strong relationships with community agencies allow navigators to liaise and refer persons with complex care needs to appropriate services; however, navigators in rural communities may struggle to connect persons to appropriate social services if these services are not available in rural areas [45].

### 4.1. Future Research

Several gaps in the literature were identified through the systematic review. First, there are opportunities for studies to examine what type of supports are essential and need to be provided by system navigators during transitions in care to improve outcomes, such as coordinating appointments or addressing barriers to care. Second, future research should explore why some studies demonstrate desirable outcomes while others do not. For example, future systematic reviews should explore whether a relationship exists between the frequency of contact between system navigators and those they are supporting, or the professional background of system navigators and the prioritized outcomes. Third, future research should explore the long-term effects of providing navigation support during transitions in care beyond the 30-day hospital readmission window. While the 30-day timeframe is frequently studied and may provide a reference for evaluating the effectiveness of interventions, a significant proportion of hospital readmissions will occur after 30 days of being discharged [28]. Finally, most studies in this review focused on navigation support provided to adults during transitions from hospital to home. Further research should examine the effectiveness of providing navigation support during transitions between non-hospital settings, such as shelters and hospices. Additionally, studies could explore which populations with complex care needs would benefit most from navigation support during transitions in care. Specifically, the expert panel noted that more research is needed to understand the effect of providing navigation support during transitions in care for children with complex care needs and their caregivers; only one study included in this review focused on children.

### 4.2. Limitations

Although this review was conducted using a comprehensive and systematic process, it was still subject to several limitations. While broad search terms were used to capture the concept of system navigators, including patient navigator, nurse navigator, and care coordinator, the term “navigation support” was not included as a key word. When formulating the review question, the expert panel originally chose the term “support from a system navigator” and synonyms for this concept were used in the search strategy. However, throughout this systematic review, the expert panel became increasingly aware that multiple titles have been used to describe the role of system navigators; therefore, the expert panel wished to focus this manuscript on the shared aspects of their role, which involves providing navigation support. While the authors believe the search was comprehensive, the omission of the term “navigation support” was a limitation. In addition, due to feasibility reasons, only peer-reviewed studies published in English from 2016 onwards were included, reference lists of included studies were not searched, and only Medline and CINAHL were searched during the update search. Potentially relevant articles may have been missed.

While the objective of this review was to analyze the effect navigation support had on priority outcomes during transitions in care for persons with complex care needs, there was heterogeneity in the interventions, which led to difficulty comparing studies. Few studies provided concrete details about the number of visits or the frequency of visits that system navigators made with patients they were supporting. As such, it was difficult to ascertain whether a relationship existed between the dose of the intervention and the reported outcomes. In addition, it was not possible to determine whether all supports listed in the studies were provided to every participant; in one study, the authors noted that supports were provided on an “as needed” basis [40]. As such, identifying which specific types of navigation support were most effective in particular circumstances was not achievable. Finally, there was variation in the tools and timeframes used to measure outcomes in the studies, such as patient QOL and patient satisfaction. Given the variation in the interventions and outcomes, it was not possible to conduct a meta-analysis.

### 4.3. Development of a Best Practice Guideline

A best practice guideline was developed by the Registered Nurses’ Association of Ontario to provide evidence-based recommendations for safe and effective transitions in care [1]. The findings from this systematic review were used to inform the development of recommendations within the guideline, specifically pertaining to navigation support during transitions in care [1]. When developing the recommendation, the expert panel used GRADE methods and considered the benefits and harms of navigation support noted in the literature, the certainty of the evidence, the preferences of persons receiving navigation support, and implications for health equity. Methods taken to develop the guideline recommendations can be found in the guideline’s appendices [1].

## 5. Conclusions

To the authors’ knowledge, this systematic review is the first to examine the impact of navigation support during transitions in care for persons with complex care needs on the outcomes of patient QOL, ED visits, follow-up visits with a health or social service provider, patient satisfaction, and readmission rates. A strength of this review was the involvement of an interprofessional expert panel that included persons with lived experience in the development of the research question and respective outcomes. Results indicate that providing navigation support during transitions in care may increase follow-up visits with a health or social service provider, reduce readmissions within 30 days of a transition in care, and increase patient satisfaction; however, the results are very uncertain. There were no important differences found regarding QOL and ED visits within 30 days of a transition in care. However, due to the very low-certainty evidence, and the degree of variation in the literature, it was difficult to draw conclusions. There is a need for more high-quality research to evaluate the effectiveness of providing navigation support during transitions in care to persons with complex care needs.

## Figures and Tables

**Table 1:** GRADE certainty of evidence.

Certainty of Evidence	Definition
High ⨁⨁⨁⨁	We are very confident that the true effect lies close to that of the estimate of the effect.
Moderate ⨁⨁⨁◯	We are moderately confident in the effect estimate. The true effect is likely to be close to the estimate of the effect, but there is a possibility that it is substantially different.
Low ⨁⨁◯◯	Our confidence in the effect estimate is limited. The true effect may be substantially different from the estimate of the effect.
Very Low ⨁◯◯◯	We have very little confidence in the effect estimate. The true effect is likely to be substantially different from the estimate of effect.

Reprinted with permission from Schunemann et al. [27]. Copyright 2013 by The GRADE Working Group.

**Table 2:** Criteria determining the certainty of the evidence.

GRADE Criteria	Definition
Risk of bias	Limitations in the study design and execution that may bias study results. Valid and reliable quality appraisal tools are used to assess the risk of bias.
Inconsistency	Unexplained differences (heterogeneity) of results across studies. Inconsistency is assessed by exploring the magnitude of difference and possible explanations in the direction and size of effects reported across studies for a defined outcome.
Indirectness	Variability between the research and review question. There are four sources of indirectness that are assessed: differences in population, interventions, outcomes measured, and comparators.
Imprecision	The degree of uncertainty around the estimate of effect. This is usually related to sample size and number of events. Studies are examined for sample size, number of events, and confidence intervals.
Publication bias	Selective publication of studies based on study results. If publication bias is strongly suspected, downgrading is considered.

Adapted with permission from Schunemann et al. [27]. Copyright 2013 by The GRADE Working Group.

## Data Availability

The original contributions presented in the study are included in the article and Supplementary Materials, further inquiries can be directed to the corresponding author.

## Supplementary Materials

The following supporting information can be downloaded at: https://www.mdpi.com/article/10.3390/healthcare12181814/s1, Table S1: PRISMA for Abstracts Checklist [26]; Table S2: PRISMA Checklist [26]. File S1. Systematic Review Search Strategy. File S2. Evidence Profile [11,12,13,14,15,16,32,33,34,35,36,37,38,39,40,41,42]. File S3. Patient Satisfaction and Quality of Life Listed in Order of Intervention Duration [12,14,32,33,34,37,39,42].
