# Supplementary material for: Navigation Support during Transitions in Care for Persons with Complex Care Needs: A Systematic Review
Source: Healthcare (Basel). 2024 Sep 10;12(18):1814. doi: 10.3390/healthcare12181814 (PMC11431248; doi:10.3390/healthcare12181814)
Supplement: Supplementary file 1 [file healthcare-12-01814-s001.zip › File S1_Systematic review search strategy.pdf]

Systematic Review Search Strategy

The systematic review search strategy was develedoped by a health science librarian. Expert panel members were also asked to review personal libraries to identify key articles not found through the search strategy, however, no additional articles were suggested by the expert panel.

The search was originally conducted for articles published between January 2016 and June 2021. An additional update search was done in January 2023 to search for articles published between June 2021 and January 2023.

Research question: Should support from a system navigator be recommended or not to improve outcomes for persons encountering a transition in care?

Population: Adult and pediatric populations with complex care needs experiencing a transition in care
Intervention: Support from a system navigator
Comparison: No support from a system navigator
Outcomes: Patient quality of life, emergency department visits (within 30 days of a transition in care), follow-up visit with a health or social service provider, patient satisfaction and readmission rates (within 30 days of a transition in care)

Inclusion Criteria:

- A primary focus on support from a system navigator provided for one week or longer following a transition in care
- A focus on pediatric or adult persons with complex care needs experiencing a transition in care:
  - Within organizations (e.g. from one hospital unit to another hospital unit), or
  - Between/across organizations or sectors (e.g. from hospital to home, home to hospice, etc.)
- A focus on one of more outcomes of interest: Patient quality of life, emergency department visits (within 30 days of a transition in care), follow-up visit with a health or social service provider, patient satisfaction or readmission rates (within 30 days of a transition in care)
- Published after January 2016
- Published in English
- Accessible for retrieval
- Peer-reviewed literature
- Any study design (e.g., quantitative, qualitative, mixed methods, systematic reviews)

Exclusion Criteria:

- Studies **NOT** related to the aforementioned interventions or prioritized outcomes
- Commentaries, editorials, narratives, case studies, pilot studies, conference abstracts, literature reviews, studies with no specific methodology
- Studies not published in English
- Upublished literature (e.g., grey literature)
- Studies published prior to 2016

Original search in 2021:

Dates searched: January 2016 – June 2021

Databases Searched: MEDLINE, MEDLINE Epub Ahead of Print and In-Process, Embase, Emcare Nursing, Cochrane Central Register of Controlled Trials, Cochrane Database of Systematic Reviews, APA PsychInfo, Cumulative Index to Nursing and Allied Health (CINAHL)

#	Database: MEDLINE	Results
1	"Continuity of Patient Care"/	19809
2	(interfacilit* adj15 (coordinat* or "co ordinat*" or collaborat*)).tw,kf.	17
3	("inter facilit*" adj15 (coordinat* or "co ordinat*" or collaborat*)).tw,kf.	8
4	(intrafacilit* adj15 (coordinat* or "co ordinat*" or collaborat*)).tw,kf.	1
5	("intra facilit*" adj15 (coordinat* or "co ordinat*" or collaborat*)).tw,kf.	0
6	(care adj5 (continuum* or continuit*)).tw,kf.	12997
7	(care adj5 (coordinat* or "co ordinat*" or collaborat*)).tw,kf.	20742
8	(healthcare adj5 (continuum* or continuit*)).tw,kf.	399
9	(interfacilit* adj5 (continuum* or continuit*)).tw,kf.	0
10	("inter facilit*" adj5 (continuum* or continuit*)).tw,kf.	0
11	(intrafacilit* adj5 (continuum* or continuit*)).tw,kf.	0
12	("intra facilit*" adj5 (continuum* or continuit*)).tw,kf.	0
13	Delivery of Health Care/	96901
14	"delivery of health care".tw,kf.	10981
15	"delivery of healthcare".tw,kf.	754
16	"health care delivery".tw,kf.	9763
17	"healthcare delivery".tw,kf.	4400
18	Delivery of Health Care, Integrated/	13392
19	(integrat* adj4 care).tw,kf.	17368
20	(integrat* adj3 healthcare).tw,kf.	2319
21	(site? adj2 care).tw,kf.	2740
22	Patient Discharge/	32396
23	(patient adj15 discharg*).tw,kf.	30682

24	(hospital adj15 discharg*).tw,kf.	60179
25	(discharg* adj15 plan*).tw,kf.	7760
26	(discharg* adj15 (home or homes)).tw,kf.	15272
27	Patient Transfer/	8924
28	(patient adj15 (transition* or transfer*)).tw,kf.	17699
29	Transitional Care/	919
30	(care adj15 (transition* or transfer*)).tw,kf.	22311
31	(healthcare adj15 (transfer* or transition*)).tw,kf.	2214
32	((facility or facilities) adj15 (transfer* or transition*)).tw,kf.	3369
33	"patient turfing*".tw,kf.	0
34	((home or homes) adj15 (transition* or transfer*)).tw,kf.	4454
35	((floor or floors) adj15 (transition* or transfer*)).tw,kf.	419
36	(setting* adj15 (transition* or transfer*)).tw,kf.	5653
37	((hospital or hospitals or intrahospital* or intrafacilit* or interfacilit* or interhospital*) adj15 transfer*).tw,kf.	11834
38	((hospital or hospitals or intrahospital* or intrafacilit* or interfacilit* or interhospital*) adj15 transition*).tw,kf.	3496
39	((shelter or shelters) adj15 (transition* or transfer*)).tw,kf.	115
40	((hospice or hospices) adj15 (transition* or transfer*)).tw,kf.	330
41	((street or streets) adj15 (transition* or transfer*)).tw,kf.	87
42	(community adj15 (transition* or transfer*)).tw,kf.	4508
43	(rehabilitation adj15 (transition* or transfer*)).tw,kf.	1987
44	or/1-43 [Care Transitions]	331848
45	Patient Navigation/	836
46	patient navigation.tw,kf.	642
47	navigator*.tw,kf.	3048
48	care coordinator*.tw,kf.	638
49	"care co ordinator*".tw,kf.	33
50	Case Managers/	198
51	case manager*.tw,kf.	3377
52	peer group/ or peer influence/	22151

53	counseling/	37019
54	social support/ or psychosocial support systems/	74171
55	52 and (53 or 54)	3416
56	(peer health mentor* or peer health coach* or peer coach* or peer mentor* or peer navigation or peer recovery coach* or peer support* or "peer to peer" or peer worker*).tw,kf.	5826
57	transition* coach*.tw,kf.	18
58	(health* coach or health* coaches or health* coaching).tw,kf.	699
59	(care coach or care coaches or care coaching).tw,kf.	32
60	Social Workers/	748
61	"social worker*".tw,kf.	9155
62	or/45-51,55-61	25510
63	44 and 62 [Care Transitions and Q1 Navigator]	4469
64	limit 63 to english language	4267
65	limit 64 to yr="2016 -Current"	1627

#	Database: MEDLINE Epub Ahead of Print and In-Process	Results
1	(interfacilit* adj15 (coordinat* or "co ordinat*" or collaborat*)).tw,kf.	3
2	("inter facilit*" adj15 (coordinat* or "co ordinat*" or collaborat*)).tw,kf.	2
3	(intrafacilit* adj15 (coordinat* or "co ordinat*" or collaborat*)).tw,kf.	0
4	("intra facilit*" adj15 (coordinat* or "co ordinat*" or collaborat*)).tw,kf.	1
5	(care adj5 (continuum* or continuit*)).tw,kf.	2422
6	(care adj5 (coordinat* or "co ordinat*" or collaborat*)).tw,kf.	4297
7	(healthcare adj5 (continuum* or continuit*)).tw,kf.	84
8	(interfacilit* adj5 (continuum* or continuit*)).tw,kf.	0
9	("inter facilit*" adj5 (continuum* or continuit*)).tw,kf.	0
10	(intrafacilit* adj5 (continuum* or continuit*)).tw,kf.	0
11	("intra facilit*" adj5 (continuum* or continuit*)).tw,kf.	0
12	"delivery of health care".tw,kf.	594
13	"delivery of healthcare".tw,kf.	233

14	"health care delivery".tw,kf.	1362
15	"healthcare delivery".tw,kf.	1068
16	(integrat* adj4 care).tw,kf.	4464
17	(integrat* adj3 healthcare).tw,kf.	572
18	(site? adj2 care).tw,kf.	431
19	(patient adj15 discharg*).tw,kf.	8129
20	(hospital adj15 discharg*).tw,kf.	9478
21	(discharg* adj15 plan*).tw,kf.	1348
22	(discharg* adj15 (home or homes)).tw,kf.	3332
23	(patient adj15 (transition* or transfer*)).tw,kf.	3527
24	(care adj15 (transition* or transfer*)).tw,kf.	5095
25	(healthcare adj15 (transfer* or transition*)).tw,kf.	661
26	((facility or facilities) adj15 (transfer* or transition*)).tw,kf.	797
27	"patient turfing*".tw,kf.	0
28	((home or homes) adj15 (transition* or transfer*)).tw,kf.	897
29	((floor or floors) adj15 (transition* or transfer*)).tw,kf.	93
30	(setting* adj15 (transition* or transfer*)).tw,kf.	1346
31	((hospital or hospitals or intrahospital* or intrafacilit* or interfacilit* or interhospital*) adj15 transfer*).tw,kf.	2184
32	((hospital or hospitals or intrahospital* or intrafacilit* or interfacilit* or interhospital*) adj15 transition*).tw,kf.	774
33	((shelter or shelters) adj15 (transition* or transfer*)).tw,kf.	31
34	((hospice or hospices) adj15 (transition* or transfer*)).tw,kf.	89
35	((street or streets) adj15 (transition* or transfer*)).tw,kf.	25
36	(community adj15 (transition* or transfer*)).tw,kf.	1008
37	(rehabilitation adj15 (transition* or transfer*)).tw,kf.	450
38	or/1-37 [Care Transitions]	40903
39	patient navigation.tw,kf.	201
40	navigator*.tw,kf.	569
41	care coordinator*.tw,kf.	137
42	"care co ordinator*".tw,kf.	13

43	case manager*.tw,kf.	285
44	(peer health mentor* or peer health coach* or peer coach* or peer mentor* or peer navigation or peer recovery coach* or peer support* or "peer to peer" or peer worker*).tw,kf.	1885
45	transition* coach*.tw,kf.	5
46	(health* coach or health* coaches or health* coaching).tw,kf.	261
47	(care coach or care coaches or care coaching).tw,kf.	10
48	"social worker*".tw,kf.	1327
49	or/39-48	4532
50	38 and 49 [Care Transitions and Q1 Navigator]	692
51	limit 50 to english language	688
52	limit 51 to yr="2016 -Current"	561

#	**Database: Embase**	**Results**
1	patient care/	314987
2	(interfacilit* adj15 (coordinat* or "co ordinat*" or collaborat*)).tw,kw.	27
3	("inter facilit*" adj15 (coordinat* or "co ordinat*" or collaborat*)).tw,kw.	20
4	(intrafacilit* adj15 (coordinat* or "co ordinat*" or collaborat*)).tw,kw.	1
5	("intra facilit*" adj15 (coordinat* or "co ordinat*" or collaborat*)).tw,kw.	1
6	(care adj5 (continuum* or continuit*)).tw,kw.	21611
7	(care adj5 (coordinat* or "co ordinat*" or collaborat*)).tw,kw.	36876
8	(healthcare adj5 (continuum* or continuit*)).tw,kw.	663
9	(interfacilit* adj5 (continuum* or continuit*)).tw,kw.	0
10	("inter facilit*" adj5 (continuum* or continuit*)).tw,kw.	0
11	(intrafacilit* adj5 (continuum* or continuit*)).tw,kw.	0
12	("intra facilit*" adj5 (continuum* or continuit*)).tw,kw.	0
13	health care delivery/	185563
14	"delivery of health care".tw,kw.	4159
15	"delivery of healthcare".tw,kw.	1267
16	"health care delivery".tw,kw.	14210

17	"healthcare delivery".tw,kw.	7480
18	integrated health care system/	11954
19	(integrat* adj4 care).tw,kw.	30193
20	(integrat* adj3 healthcare).tw,kw.	4318
21	(site? adj2 care).tw,kw.	4703
22	Patient Discharge/	115277
23	(patient adj15 discharg*).tw,kw.	80807
24	(hospital adj15 discharg*).tw,kw.	118110
25	(discharg* adj15 plan*).tw,kw.	15565
26	(discharg* adj15 (home or homes)).tw,kw.	40032
27	patient transport/	29078
28	(patient adj15 (transition* or transfer*)).tw,kw.	40488
29	transitional care/	3676
30	(care adj15 (transition* or transfer*)).tw,kw.	46777
31	(healthcare adj15 (transfer* or transition*)).tw,kw.	4554
32	((facility or facilities) adj15 (transfer* or transition*)).tw,kw.	7789
33	"patient turfing*".tw,kw.	0
34	((home or homes) adj15 (transition* or transfer*)).tw,kw.	8978
35	((floor or floors) adj15 (transition* or transfer*)).tw,kw.	1227
36	(setting* adj15 (transition* or transfer*)).tw,kw.	11045
37	((hospital or hospitals or intrahospital* or intrafacilit* or interfacilit* or interhospital*) adj15 transfer*).tw,kw.	25717
38	((hospital or hospitals or intrahospital* or intrafacilit* or interfacilit* or interhospital*) adj15 transition*).tw,kw.	7362
39	((shelter or shelters) adj15 (transition* or transfer*)).tw,kw.	196
40	((hospice or hospices) adj15 (transition* or transfer*)).tw,kw.	1182
41	((street or streets) adj15 (transition* or transfer*)).tw,kw.	182
42	(community adj15 (transition* or transfer*)).tw,kw.	7697
43	(rehabilitation adj15 (transition* or transfer*)).tw,kw.	4764
44	or/1-43 [Care Transitions]	908328
45	patient navigation.tw,kw.	1472

46	navigator*.tw,kw.	6273
47	care coordinator/	332
48	care coordinator*.tw,kw.	1374
49	"care co ordinator*".tw,kw.	67
50	case manager/	2074
51	case manager*.tw,kw.	5016
52	exp peer group/	26200
53	counseling/	74356
54	psychosocial care/ or social support/	116363
55	52 and (53 or 54)	3804
56	peer counseling/	619
57	(peer health mentor* or peer health coach* or peer coach* or peer mentor* or peer navigation or peer recovery coach* or peer support* or "peer to peer" or peer worker*).tw,kw.	10807
58	transition* coach*.tw,kw.	45
59	(health* coach or health* coaches or health* coaching).tw,kw.	1476
60	(care coach or care coaches or care coaching).tw,kw.	76
61	social worker/	14067
62	"social worker*".tw,kw.	18349
63	or/45-51,55-62	50013
64	44 and 63 [Care Transitions and Q1 Navigator]	12851
65	limit 64 to english language	12393
66	limit 65 to yr="2016 -Current"	5378
67	limit 66 to (books or chapter or conference abstract or conference paper or "conference review" or editorial)	2309
68	66 not 67	3069

#	**Database: Emcare Nursing**	**Results**
1	patient care/	141908
2	(interfacilit* adj15 (coordinat* or "co ordinat*" or collaborat*)).tw,kw.	12

3	("inter facilit*" adj15 (coordinat* or "co ordinat*" or collaborat*)).tw,kw.	6
4	(intrafacilit* adj15 (coordinat* or "co ordinat*" or collaborat*)).tw,kw.	1
5	("intra facilit*" adj15 (coordinat* or "co ordinat*" or collaborat*)).tw,kw.	1
6	(care adj5 (continuum* or continuit*)).tw,kw.	10801
7	(care adj5 (coordinat* or "co ordinat*" or collaborat*)).tw,kw.	18017
8	(healthcare adj5 (continuum* or continuit*)).tw,kw.	359
9	(interfacilit* adj5 (continuum* or continuit*)).tw,kw.	0
10	("inter facilit*" adj5 (continuum* or continuit*)).tw,kw.	0
11	(intrafacilit* adj5 (continuum* or continuit*)).tw,kw.	0
12	("intra facilit*" adj5 (continuum* or continuit*)).tw,kw.	0
13	health care delivery/	68512
14	"delivery of health care".tw,kw.	2208
15	"delivery of healthcare".tw,kw.	732
16	"health care delivery".tw,kw.	6095
17	"healthcare delivery".tw,kw.	3658
18	integrated health care system/	3323
19	(integrat* adj4 care).tw,kw.	15371
20	(integrat* adj3 healthcare).tw,kw.	2007
21	(site? adj2 care).tw,kw.	2007
22	Patient Discharge/	28385
23	(patient adj15 discharg*).tw,kw.	16949
24	(hospital adj15 discharg*).tw,kw.	33249
25	(discharg* adj15 plan*).tw,kw.	4222
26	(discharg* adj15 (home or homes)).tw,kw.	10271
27	patient transport/	12601
28	(patient adj15 (transition* or transfer*)).tw,kw.	9812
29	transitional care/	1698
30	(care adj15 (transition* or transfer*)).tw,kw.	18103
31	(healthcare adj15 (transfer* or transition*)).tw,kw.	2053
32	((facility or facilities) adj15 (transfer* or transition*)).tw,kw.	2191
33	"patient turfing*".tw,kw.	0

34	((home or homes) adj15 (transition* or transfer*)).tw,kw.	3734
35	((floor or floors) adj15 (transition* or transfer*)).tw,kw.	222
36	(setting* adj15 (transition* or transfer*)).tw,kw.	4128
37	((hospital or hospitals or intrahospital* or intrafacilit* or interfacilit* or interhospital*) adj15 transfer*).tw,kw.	6693
38	((hospital or hospitals or intrahospital* or intrafacilit* or interfacilit* or interhospital*) adj15 transition*).tw,kw.	2840
39	((shelter or shelters) adj15 (transition* or transfer*)).tw,kw.	103
40	((hospice or hospices) adj15 (transition* or transfer*)).tw,kw.	294
41	((street or streets) adj15 (transition* or transfer*)).tw,kw.	85
42	(community adj15 (transition* or transfer*)).tw,kw.	3285
43	(rehabilitation adj15 (transition* or transfer*)).tw,kw.	1735
44	or/1-43 [Care Transitions]	326166
45	patient navigation.tw,kw.	568
46	navigator*.tw,kw.	2277
47	care coordinator/	188
48	care coordinator*.tw,kw.	605
49	"care co ordinator*".tw,kw.	43
50	case manager/	2521
51	case manager*.tw,kw.	2745
52	exp peer group/	12533
53	counseling/	38245
54	psychosocial care/ or social support/	61176
55	52 and (53 or 54)	1539
56	peer counseling/	500
57	(peer health mentor* or peer health coach* or peer coach* or peer mentor* or peer navigation or peer recovery coach* or peer support* or "peer to peer" or peer worker*).tw,kw.	6416
58	transition* coach*.tw,kw.	18
59	(health* coach or health* coaches or health* coaching).tw,kw.	670
60	(care coach or care coaches or care coaching).tw,kw.	28

61	social worker/	10347
62	"social worker*".tw,kw.	11056
63	or/45-51,55-62	27253
64	44 and 63 [Care Transitions and Q1 Navigator]	5516
65	limit 64 to english language	5401
66	limit 65 to yr="2016 -Current"	2187
67	limit 66 to (books or chapter or conference abstract or conference paper or "conference review" or dissertation or editorial)	52
68	66 not 67	2135

Database: Cochrane Central Register of Controlled Trials
Search strategy:

#	Searches	Results
1	"Continuity of Patient Care"/	627
2	(interfacilit* adj15 (coordinat* or "co ordinat*" or collaborat*)).tw,kw.	1
3	("inter facilit*" adj15 (coordinat* or "co ordinat*" or collaborat*)).tw,kw.	0
4	(intrafacilit* adj15 (coordinat* or "co ordinat*" or collaborat*)).tw,kw.	0
5	("intra facilit*" adj15 (coordinat* or "co ordinat*" or collaborat*)).tw,kw.	0
6	(care adj5 (continuum* or continuit*)).tw,kw.	1148
7	(care adj5 (coordinat* or "co ordinat*" or collaborat*)).tw,kw.	3790
8	(healthcare adj5 (continuum* or continuit*)).tw,kw.	43
9	(interfacilit* adj5 (continuum* or continuit*)).tw,kw.	0
10	("inter facilit*" adj5 (continuum* or continuit*)).tw,kw.	0
11	(intrafacilit* adj5 (continuum* or continuit*)).tw,kw.	0
12	("intra facilit*" adj5 (continuum* or continuit*)).tw,kw.	0
13	Delivery of Health Care/	791
14	"delivery of health care".tw,kw.	47
15	"delivery of healthcare".tw,kw.	28
16	"health care delivery".tw,kw.	1859
17	"healthcare delivery".tw,kw.	255

18	Delivery of Health Care, Integrated/	397
19	(integrat* adj4 care).tw,kw.	3420
20	(integrat* adj3 healthcare).tw,kw.	263
21	(site? adj2 care).tw,kw.	992
22	Patient Discharge/	1554
23	(patient adj15 discharg*).tw,kw.	7463
24	(hospital adj15 discharg*).tw,kw.	16365
25	(discharg* adj15 plan*).tw,kw.	1304
26	(discharg* adj15 (home or homes)).tw,kw.	3981
27	Patient Transfer/	149
28	(patient adj15 (transition* or transfer*)).tw,kw.	3035
29	Transitional Care/	58
30	(care adj15 (transition* or transfer*)).tw,kw.	3579
31	(healthcare adj15 (transfer* or transition*)).tw,kw.	299
32	((facility or facilities) adj15 (transfer* or transition*)).tw,kw.	331
33	"patient turfing*".tw,kw.	0
34	((home or homes) adj15 (transition* or transfer*)).tw,kw.	965
35	((floor or floors) adj15 (transition* or transfer*)).tw,kw.	67
36	(setting* adj15 (transition* or transfer*)).tw,kw.	1048
37	((hospital or hospitals or intrahospital* or intrafacilit* or interfacilit* or interhospital*) adj15 transfer*).tw,kw.	1459
38	((hospital or hospitals or intrahospital* or intrafacilit* or interfacilit* or interhospital*) adj15 transition*).tw,kw.	744
39	((shelter or shelters) adj15 (transition* or transfer*)).tw,kw.	18
40	((hospice or hospices) adj15 (transition* or transfer*)).tw,kw.	42
41	((street or streets) adj15 (transition* or transfer*)).tw,kw.	10
42	(community adj15 (transition* or transfer*)).tw,kw.	697
43	(rehabilitation adj15 (transition* or transfer*)).tw,kw.	421
44	or/1-43 [Care Transitions]	39427
45	Patient Navigation/	129
46	patient navigation.tw,kw.	379

47	navigator*.tw,kw.	813
48	care coordinator*.tw,kw.	218
49	care coordinator*.tw,kw.	218
50	case manager*.tw,kw.	757
51	peer group/	1415
52	counseling/	4260
53	social support/	3374
54	51 and (52 or 53)	337
55	(peer health mentor* or peer health coach* or peer coach* or peer mentor* or peer navigation or peer recovery coach* or peer support* or "peer to peer" or peer worker*).tw,kw.	1874
56	transition* coach*.tw,kw.	13
57	(health* coach or health* coaches or health* coaching).tw,kw.	776
58	(care coach or care coaches or care coaching).tw,kw.	55
59	"social worker*".tw,kw.	1184
60	or/45-50,54-59 [Care Transitions and Q1 Navigator]	5817
61	44 and 60	1286
62	limit 61 to english language	749
63	limit 62 to yr="2016 -Current"	440

#	**Database: Cochrane Database of Systematic Reviews**	**Results**
1	(interfacilit* adj15 (coordinat* or "co ordinat*" or collaborat*)).tw,kw.	0
2	("inter facilit*" adj15 (coordinat* or "co ordinat*" or collaborat*)).tw,kw.	0
3	(intrafacilit* adj15 (coordinat* or "co ordinat*" or collaborat*)).tw,kw.	0
4	("intra facilit*" adj15 (coordinat* or "co ordinat*" or collaborat*)).tw,kw.	0
5	(care adj5 (continuum* or continuit*)).tw,kw.	141
6	(care adj5 (coordinat* or "co ordinat*" or collaborat*)).tw,kw.	239
7	(healthcare adj5 (continuum* or continuit*)).tw,kw.	8
8	(interfacilit* adj5 (continuum* or continuit*)).tw,kw.	0
9	("inter facilit*" adj5 (continuum* or continuit*)).tw,kw.	0

10	(intrafacilit* adj5 (continuum* or continuit*)).tw,kw.	0
11	("intra facilit*" adj5 (continuum* or continuit*)).tw,kw.	0
12	"delivery of health care".tw,kw.	69
13	"delivery of healthcare".tw,kw.	125
14	"health care delivery".tw,kw.	41
15	"healthcare delivery".tw,kw.	93
16	(integrat* adj4 care).tw,kw.	166
17	(integrat* adj3 healthcare).tw,kw.	23
18	(site? adj2 care).tw,kw.	37
19	(patient adj15 discharg*).tw,kw.	225
20	(hospital adj15 discharg*).tw,kw.	1144
21	(discharg* adj15 plan*).tw,kw.	108
22	(discharg* adj15 (home or homes)).tw,kw.	250
23	(patient adj15 (transition* or transfer*)).tw,kw.	135
24	(care adj15 (transition* or transfer*)).tw,kw.	270
25	(healthcare adj15 (transfer* or transition*)).tw,kw.	57
26	((facility or facilities) adj15 (transfer* or transition*)).tw,kw.	65
27	"patient turfing*".tw,kw.	0
28	((home or homes) adj15 (transition* or transfer*)).tw,kw.	61
29	((floor or floors) adj15 (transition* or transfer*)).tw,kw.	5
30	(setting* adj15 (transition* or transfer*)).tw,kw.	126
31	((hospital or hospitals or intrahospital* or intrafacilit* or interfacilit* or interhospital*) adj15 transfer*).tw,kw.	137
32	((hospital or hospitals or intrahospital* or intrafacilit* or interfacilit* or interhospital*) adj15 transition*).tw,kw.	40
33	((shelter or shelters) adj15 (transition* or transfer*)).tw,kw.	3
34	((hospice or hospices) adj15 (transition* or transfer*)).tw,kw.	4
35	((street or streets) adj15 (transition* or transfer*)).tw,kw.	2
36	(community adj15 (transition* or transfer*)).tw,kw.	62
37	(rehabilitation adj15 (transition* or transfer*)).tw,kw.	37
38	or/1-37 [Care Transitions]	2002

39	patient navigation.tw,kw.	1
40	navigator*.tw,kw.	28
41	care coordinator*.tw,kw.	8
42	care coordinator*.tw,kw.	8
43	case manager*.tw,kw.	77
44	(peer health mentor* or peer health coach* or peer coach* or peer mentor* or peer navigation or peer recovery coach* or peer support* or "peer to peer" or peer worker*).tw,kw.	163
45	transition* coach*.tw,kw.	0
46	(health* coach or health* coaches or health* coaching).tw,kw.	13
47	(care coach or care coaches or care coaching).tw,kw.	1
48	"social worker*".tw,kw.	214
49	or/39-48 [Care Transitions and Q1 Navigator]	425
50	38 and 49	232
51	limit 50 to last 5 years	95

#	APA PsychInfo	Results
1	"continuum of care"/	1949
2	(interfacilit* adj15 (coordinat* or "co ordinat*" or collaborat*)).tw,id.	1
3	("inter facilit*" adj15 (coordinat* or "co ordinat*" or collaborat*)).tw,id.	1
4	(intrafacilit* adj15 (coordinat* or "co ordinat*" or collaborat*)).tw,id.	0
5	("intra facilit*" adj15 (coordinat* or "co ordinat*" or collaborat*)).tw,id.	1
6	(care adj5 (continuum* or continuit*)).tw,id.	4898
7	(care adj5 (coordinat* or "co ordinat*" or collaborat*)).tw,id.	8552
8	(healthcare adj5 (continuum* or continuit*)).tw,id.	108
9	(interfacilit* adj5 (continuum* or continuit*)).tw,id.	0
10	("inter facilit*" adj5 (continuum* or continuit*)).tw,id.	0
11	(intrafacilit* adj5 (continuum* or continuit*)).tw,id.	0
12	("intra facilit*" adj5 (continuum* or continuit*)).tw,id.	0
13	health care delivery/	21286

14	"delivery of health care".tw,id.	684
15	"delivery of healthcare".tw,id.	202
16	"health care delivery".tw,id.	5166
17	"healthcare delivery".tw,id.	1110
18	(integrat* adj4 care).tw,id.	7656
19	(integrat* adj3 healthcare).tw,id.	606
20	(site? adj2 care).tw,id.	743
21	hospital discharge/ or facility discharge/	2729
22	(patient adj15 discharg*).tw,id.	3373
23	((hospital or facility) adj15 discharg*).tw,id.	9078
24	(discharg* adj15 plan*).tw,id.	1890
25	(discharg* adj15 (home or homes)).tw,id.	2260
26	client transfer/	268
27	((patient or client) adj15 (transition* or transfer*)).tw,id.	5715
28	(care adj15 (transition* or transfer*)).tw,id.	7481
29	(healthcare adj15 (transfer* or transition*)).tw,id.	744
30	((facility or facilities) adj15 (transfer* or transition*)).tw,id.	860
31	"patient turfing*".tw,id.	0
32	((home or homes) adj15 (transition* or transfer*)).tw,id.	2830
33	((floor or floors) adj15 (transition* or transfer*)).tw,id.	72
34	(setting* adj15 (transition* or transfer*)).tw,id.	3824
35	((hospital or hospitals or intrahospital* or intrafacilit* or interfacilit* or interhospital*) adj15 transfer*).tw,id.	1380
36	((hospital or hospitals or intrahospital* or intrafacilit* or interfacilit* or interhospital*) adj15 transition*).tw,id.	1190
37	((shelter or shelters) adj15 (transition* or transfer*)).tw,id.	187
38	((hospice or hospices) adj15 (transition* or transfer*)).tw,id.	142
39	((street or streets) adj15 (transition* or transfer*)).tw,id.	142
40	(community adj15 (transition* or transfer*)).tw,id.	4196
41	(rehabilitation adj15 (transition* or transfer*)).tw,id.	981
42	or/1-41 [Care Transitions]	75362

43	patient navigation.tw,id.	258
44	navigator*.tw,id.	919
45	care coordinator*.tw,id.	295
46	"care co ordinator*".tw,id.	28
47	case manager*.tw,id.	2174
48	peers/ or peer relations/ or peer pressure/	30200
49	counseling/	23895
50	social support/	38337
51	coaching/ or coaches/ or life coaching/	7551
52	48 and (49 or 50 or 51)	1739
53	peer counseling/	1156
54	(peer health mentor* or peer health coach* or peer coach* or peer mentor* or peer navigation or peer recovery coach* or peer support* or "peer to peer" or peer worker*).tw,id.	7765
55	transition* coach*.tw,id.	18
56	(health* coach or health* coaches or health* coaching).tw,id.	294
57	(care coach or care coaches or care coaching).tw,id.	7
58	social workers/ or psychiatric social workers/	13019
59	social workers/ or psychiatric social workers/	13019
60	"social worker*".tw,id.	25942
61	or/43-47,52-60	41914
62	42 and 61 [Care Transitions and Q1 Navigator]	2830
63	limit 62 to english language	2712
64	limit 63 to yr="2016 -Current"	801
65	limit 64 to ("0200 book" or "0240 authored book" or "0280 edited book" or "0300 encyclopedia" or "0400 dissertation abstract")	189
66	64 not 65	612

	**Database: Cumulative Index to Nursing and Allied Health (CINAHL)**		
#	Query	Limiters/Expanders	Results

S1	(MH "Continuity of Patient Care")	Expanders - Apply equivalent subjects<br>Search modes - Boolean/Phrase	15,440
S2	TI (interfacilit* N15 (coordinat* OR "co ordinat*")) OR AB (interfacilit* N15 (coordinat* OR "co ordinat*"))	Expanders - Apply equivalent subjects<br>Search modes - Boolean/Phrase	7
S3	TI (inter facilit* N15 (coordinat* OR "co ordinat*")) OR AB (inter facilit* N15 (coordinat* OR "co ordinat*"))	Expanders - Apply equivalent subjects<br>Search modes - Boolean/Phrase	4
S4	TI (intrafacilit* N15 (coordinat* OR "co ordinat*")) OR AB (intra facilit* N15 (coordinat* OR "co ordinat*"))	Expanders - Apply equivalent subjects<br>Search modes - Boolean/Phrase	0
S5	TI (intra facilit* N15 (coordinat* OR "co ordinat*")) OR AB (intra facilit* N15 (coordinat* OR "co ordinat*"))	Expanders - Apply equivalent subjects<br>Search modes - Boolean/Phrase	0
S6	TI (care N5 (continuum* OR continuit*)) OR AB (care N5 (continuum* OR continuit*))	Expanders - Apply equivalent subjects<br>Search modes - Boolean/Phrase	10,754
S7	TI (care N5 (coordinat* OR "co ordinat*")) OR AB (care N5 (coordinat* OR "co ordinat*"))	Expanders - Apply equivalent subjects<br>Search modes - Boolean/Phrase	10,996
S8	TI (healthcare N5 (continuum* OR continuit*)) OR AB (healthcare N5 (continuum* OR continuit*))	Expanders - Apply equivalent subjects<br>Search modes - Boolean/Phrase	1,116
S9	TI (interfacilt* N5 (continuum* OR continuit*)) OR AB (interfacilt* N5 (continuum* OR continuit*))	Expanders - Apply equivalent subjects<br>Search modes - Boolean/Phrase	0
S10	TI (inter facilt* N5 (continuum* OR continuit*)) OR AB (inter facilt* N5 (continuum* OR continuit*))	Expanders - Apply equivalent subjects<br>Search modes - Boolean/Phrase	0
S11	TI (intrafacilt* N5 (continuum* OR continuit*)) OR AB (intrafacilt* N5 (continuum* OR continuit*))	Expanders - Apply equivalent subjects<br>Search modes - Boolean/Phrase	0
S12	TI (intra facilt* N5 (continuum* OR continuit*)) OR AB (intra facilt* N5 (continuum* OR continuit*))	Expanders - Apply equivalent subjects<br>Search modes - Boolean/Phrase	0
S13	TI (healthcare N5 (coordinat* OR "co ordinat*")) OR AB (healthcare N5 (coordinat* OR "co ordinat*"))	Expanders - Apply equivalent subjects<br>Search modes - Boolean/Phrase	1,814
S14	TI (healthcare N5 (continuum* OR continuit*)) OR AB (healthcare N5 (continuum* OR continuit*))	Expanders - Apply equivalent subjects<br>Search modes - Boolean/Phrase	1,116
S15	(MH "Health Care Delivery")	Expanders - Apply equivalent subjects<br>Search modes - Boolean/Phrase	55,711

S16	TI "delivery of health care" OR AB "delivery of health care"	Expanders - Apply equivalent subjects<br>Search modes - Boolean/Phrase	1,033
S17	TI "delivery of healthcare" OR AB "delivery of healthcare"	Expanders - Apply equivalent subjects<br>Search modes - Boolean/Phrase	488
S18	TI "health care delivery" OR AB "health care delivery"	Expanders - Apply equivalent subjects<br>Search modes - Boolean/Phrase	5,495
S19	TI "healthcare delivery" OR AB "healthcare delivery"	Expanders - Apply equivalent subjects<br>Search modes - Boolean/Phrase	3,209
S20	(MH "Health Care Delivery, Integrated")	Expanders - Apply equivalent subjects<br>Search modes - Boolean/Phrase	12,601
S21	TI (integrat* N3 (care OR healthcare)) OR AB (integrat* N3 (care OR healthcare))	Expanders - Apply equivalent subjects<br>Search modes - Boolean/Phrase	18,063
S22	TI (site# N2 care) OR AB (site# N2 care)	Expanders - Apply equivalent subjects<br>Search modes - Boolean/Phrase	2,595
S23	(MH "Early Patient Discharge") OR (MH "Patient Discharge")	Expanders - Apply equivalent subjects<br>Search modes - Boolean/Phrase	20,884
S24	TI (patient N15 discharg*) OR AB (patient N15 discharg*)	Expanders - Apply equivalent subjects<br>Search modes - Boolean/Phrase	41,796
S25	TI (hospital N15 discharge*) OR AB (hospital N15 discharge*)	Expanders - Apply equivalent subjects<br>Search modes - Boolean/Phrase	30,113
S26	TI (discharg* N15 (home or homes)) OR AB (discharg* N15 (home or homes))	Expanders - Apply equivalent subjects<br>Search modes - Boolean/Phrase	9,238
S27	(MH "Discharge Planning")	Expanders - Apply equivalent subjects<br>Search modes - Boolean/Phrase	5,300
S28	TI (discharg* N15 plan*) OR AB (discharg* N15 plan*)	Expanders - Apply equivalent subjects<br>Search modes - Boolean/Phrase	4,871
S29	(MH "Transfer, Discharge")	Expanders - Apply equivalent subjects<br>Search modes - Boolean/Phrase	6,133
S30	TI (patient* N15 (transition* OR transfer*)) OR AB (patient* N15 (transition* OR transfer*))	Expanders - Apply equivalent subjects<br>Search modes - Boolean/Phrase	21,352

S31	(MH "Transitional Care")	Expanders - Apply equivalent subjects<br>Search modes - Boolean/Phrase	2,079
S32	TI (care N15 (transition* or transfer*)) OR AB (care N15 (transition* or transfer*))	Expanders - Apply equivalent subjects<br>Search modes - Boolean/Phrase	18,122
S33	TI (healthcare N15 (transfer* or transition*)) OR AB (healthcare N15 (transfer* or transition*))	Expanders - Apply equivalent subjects<br>Search modes - Boolean/Phrase	4,896
S34	TI (facility N15 (transfer* OR transition*)) OR AB (facility N15 (transfer* OR transition*))	Expanders - Apply equivalent subjects<br>Search modes - Boolean/Phrase	1,993
S35	TI (facilities N15 (transfer* OR transition*)) OR AB (facilities N15 (transfer* OR transition*))	Expanders - Apply equivalent subjects<br>Search modes - Boolean/Phrase	1,993
S36	TI ("patient turfing*") OR AB ("patient turfing*")	Expanders - Apply equivalent subjects<br>Search modes - Boolean/Phrase	0
S37	TI (home N15 (transition* or transfer*)) OR AB (home N15 (transition* or transfer*))	Expanders - Apply equivalent subjects<br>Search modes - Boolean/Phrase	3,985
S38	TI (homes N15 (transition* or transfer*)) OR AB (homes N15 (transition* or transfer*))	Expanders - Apply equivalent subjects<br>Search modes - Boolean/Phrase	3,985
S39	TI (floor N15 (transition* or transfer*)) OR AB (floor N15 (transition* or transfer*))	Expanders - Apply equivalent subjects<br>Search modes - Boolean/Phrase	178
S40	TI (setting* N15 (transition* or transfer*)) OR AB (setting* N15 (transition* or transfer*))	Expanders - Apply equivalent subjects<br>Search modes - Boolean/Phrase	3,741
S41	(MH "Transfer, Intrahospital")	Expanders - Apply equivalent subjects<br>Search modes - Boolean/Phrase	1,272
S42	TI ((hospital or hospitals or intrahospital* or intrafacilit* or interfacilit* or interhospital*) N15 transfer*) OR AB ((hospital or hospitals or intrahospital* or intrafacilit* or interfacilit* or interhospital*) N15 transfer*)	Expanders - Apply equivalent subjects<br>Search modes - Boolean/Phrase	5,487
S43	TI ((hospital or hospitals or intrahospital* or intrafacilit* or interfacilit* or interhospital*) N15 transition*) OR AB ((hospital or hospitals or intrahospital* or intrafacilit* or interfacilit* or interhospital*) N15 transition*)	Expanders - Apply equivalent subjects<br>Search modes - Boolean/Phrase	3,289
S44	TI ((shelter or shelters) N15 (transition* or transfer*)) OR AB ((shelter or shelters) N15 (transition* or transfer*))	Expanders - Apply equivalent subjects<br>Search modes - Boolean/Phrase	98

S45	TI ((hospice or hospices) N15 (transition* or transfer*)) OR AB ((hospice or hospices) N15 (transition* or transfer*))	Expanders - Apply equivalent subjects<br>Search modes - Boolean/Phrase	349
S46	TI ((street or streets) N15 (transition* or transfer*)) OR AB ((street or streets) N15 (transition* or transfer*))	Expanders - Apply equivalent subjects<br>Search modes - Boolean/Phrase	62
S47	TI (community N15 (transition* or transfer*)) OR AB (community N15 (transition* or transfer*))	Expanders - Apply equivalent subjects<br>Search modes - Boolean/Phrase	3,497
S48	TI (rehabilitation N15 (transition* or transfer*)) OR AB (rehabilitation N15 (transition* or transfer*))	Expanders - Apply equivalent subjects<br>Search modes - Boolean/Phrase	1,557
S49	TI (system N5 navigat*) OR AB (system N5 navigat*)	Expanders - Apply equivalent subjects<br>Search modes - Boolean/Phrase	2,723
S50	S1 OR S2 OR S3 OR S4 OR S5 OR S6 OR S7 OR S8 OR S9 OR S10 OR S11 OR S12 OR S13 OR S14 OR S15 OR S16 OR S17 OR S18 OR S19 OR S20 OR S21 OR S22 OR S23 OR S24 OR S25 OR S26 OR S27 OR S28 OR S29 OR S30 OR S31 OR S32 OR S33 OR S34 OR S35 OR S36 OR S37 OR S38 OR S39 OR S40 OR S41 OR S42 OR S43 OR S44 OR S45 OR S46 OR S47 OR S48 OR S49	Expanders - Apply equivalent subjects<br>Search modes - Boolean/Phrase	220,322
S51	(MH "Patient Navigation")	Expanders - Apply equivalent subjects<br>Search modes - Boolean/Phrase	1,502
S52	TI ("patient navigation") OR AB ("patient navigation")	Expanders - Apply equivalent subjects<br>Search modes - Boolean/Phrase	630
S53	TI navigator* OR AB navigator*	Expanders - Apply equivalent subjects<br>Search modes - Boolean/Phrase	2,055
S54	TI "care coordinator*" OR AB "care coordinator*"	Expanders - Apply equivalent subjects<br>Search modes - Boolean/Phrase	724
S55	TI ("care co ordinator*") OR AB ("care co ordinator*")	Expanders - Apply equivalent subjects<br>Search modes - Boolean/Phrase	65
S56	(MH "Case Managers")	Expanders - Apply equivalent subjects<br>Search modes - Boolean/Phrase	4,159
S57	TI "case manager*" OR AB "case manager*"	Expanders - Apply equivalent subjects<br>Search modes - Boolean/Phrase	4,453

S58	(MH "Peer Group")	Expanders - Apply equivalent subjects<br>Search modes - Boolean/Phrase	14,024
S59	(MH "Counseling")	Expanders - Apply equivalent subjects<br>Search modes - Boolean/Phrase	31,841
S60	(MH "Support, Psychosocial")	Expanders - Apply equivalent subjects<br>Search modes - Boolean/Phrase	84,710
S61	S58 AND (S59 OR S60)	Expanders - Apply equivalent subjects<br>Search modes - Boolean/Phrase	2,827
S62	(MH "Peer Assistance Programs")	Expanders - Apply equivalent subjects<br>Search modes - Boolean/Phrase	951
S63	TI ("peer health mentor*" or "peer health coach*" or "peer coach*" or "peer mentor*" or "peer navigation" or "peer recovery coach*" or "peer support*" or "peer to peer" or "peer worker*") OR AB ("peer health mentor*" or "peer health coach*" or "peer coach*" or "peer mentor*" or "peer navigation" or "peer recovery coach*" or "peer support*" or "peer to peer" or "peer worker*")	Expanders - Apply equivalent subjects<br>Search modes - Boolean/Phrase	6,005
S64	TI "transition* coach*" OR AB "transition* coach*"	Expanders - Apply equivalent subjects<br>Search modes - Boolean/Phrase	27
S65	TI ("health* coach" or "health* coaches" or "health* coaching") OR AB ("health* coach" or "health* coaches" or "health* coaching")	Expanders - Apply equivalent subjects<br>Search modes - Boolean/Phrase	754
S66	TI ("care coach" or "care coaches" or "care coaching") OR AB ("care coach" or "care coaches" or "care coaching")	Expanders - Apply equivalent subjects<br>Search modes - Boolean/Phrase	37
S67	(MH "Social Workers")	Expanders - Apply equivalent subjects<br>Search modes - Boolean/Phrase	9,566
S68	TI "social worker*" OR AB "social worker*"	Expanders - Apply equivalent subjects<br>Search modes - Boolean/Phrase	11,470
S69	S51 OR S52 OR S53 OR S54 OR S55 OR S56 OR S57 OR S61 OR S62 OR S63 OR S64 OR S65 OR S66 OR S67 OR S68	Expanders - Apply equivalent subjects<br>Search modes - Boolean/Phrase	36,488
S70	S50 AND S69	Expanders - Apply equivalent subjects<br>Search modes - Boolean/Phrase	5,778
S71	S50 AND S69	Limiters - Peer Reviewed<br>Expanders - Apply equivalent	4,508

		subjects<br>Search modes - Boolean/Phrase	
S72	S50 AND S69	Limiters - Published Date: 20160101-20211231; Peer Reviewed<br>Expanders - Apply equivalent subjects<br>Search modes - Boolean/Phrase	2,281
S73	S50 AND S69	Limiters - Published Date: 20160101-20211231; Peer Reviewed<br>Expanders - Apply equivalent subjects<br>Search modes - Boolean/Phrase	2,281

Update Search in 2023:

Dates searched: June 2021 – January 2023

Databases Searched: CINAHL and MEDLINE

**Notes: The same original search strategy and same inclusion and exclusion criteria were used to complete the update search in 2023 but the search was only applied to CINAHL and MEDLINE for feasibility purposes.*

The librarian also found that a new subject heading had been created that was not available at the time the original search was conducted in 2021. This subject heading and the associated textword string was added to the search strategy for the update search.

- *Newly added subject heading: MeSH term Hospital to Home Transition/*
- *Associated textword string: ("hospital to home" or "hospital to homes" or "H2H transition*")*

#	Database: Medline	Results
1	"Continuity of Patient Care"/	20540
2	(interfacilit* adj15 (coordinat* or "co ordinat*" or collaborat*)).tw,kf.	21
3	("inter facilit*" adj15 (coordinat* or "co ordinat*" or collaborat*)).tw,kf.	13
4	(intrafacilit* adj15 (coordinat* or "co ordinat*" or collaborat*)).tw,kf.	1
5	("intra facilit*" adj15 (coordinat* or "co ordinat*" or collaborat*)).tw,kf.	1
6	(care adj5 (continuum* or continuit*)).tw,kf.	18162
7	(care adj5 (coordinat* or "co ordinat*" or collaborat*)).tw,kf.	29180
8	(healthcare adj5 (continuum* or continuit*)).tw,kf.	614
9	(interfacilit* adj5 (continuum* or continuit*)).tw,kf.	0
10	("inter facilit*" adj5 (continuum* or continuit*)).tw,kf.	0
11	(intrafacilit* adj5 (continuum* or continuit*)).tw,kf.	0
12	("intra facilit*" adj5 (continuum* or continuit*)).tw,kf.	0
13	Delivery of Health Care/	113469
14	"delivery of health care".tw,kf.	12178
15	"delivery of healthcare".tw,kf.	1295
16	"health care delivery".tw,kf.	12118
17	"healthcare delivery".tw,kf.	6679
18	Delivery of Health Care, Integrated/	14155
19	(integrat* adj4 care).tw,kf.	26183
20	(integrat* adj3 healthcare).tw,kf.	3608
21	(site? adj2 care).tw,kf.	3568
22	Patient Discharge/	38271
23	(patient adj15 discharg*).tw,kf.	45050
24	(hospital adj15 discharg*).tw,kf.	79535
25	(discharg* adj15 plan*).tw,kf.	10367
26	(discharg* adj15 (home or homes)).tw,kf.	21552

27	Patient Transfer/	9551
28	(patient adj15 (transition* or transfer*)).tw,kf.	24150
29	Transitional Care/	1200
30	(care adj15 (transition* or transfer*)).tw,kf.	32216
31	(healthcare adj15 (transfer* or transition*)).tw,kf.	3693
32	((facility or facilities) adj15 (transfer* or transition*)).tw,kf.	4792
33	"patient turfing*".tw,kf.	0
34	Hospital to Home Transition/	34
35	("hospital to home" or "hospital to homes" or "H2H transition*").tw,kf.	3507
36	((home or homes) adj15 (transition* or transfer*)).tw,kf.	6239
37	((floor or floors) adj15 (transition* or transfer*)).tw,kf.	586
38	(setting* adj15 (transition* or transfer*)).tw,kf.	8215
39	((hospital or hospitals or intrahospital* or intrafacilit* or interfacilit* or interhospital*) adj15 transfer*).tw,kf.	16072
40	((hospital or hospitals or intrahospital* or intrafacilit* or interfacilit* or interhospital*) adj15 transition*).tw,kf.	5071
41	((shelter or shelters) adj15 (transition* or transfer*)).tw,kf.	175
42	((hospice or hospices) adj15 (transition* or transfer*)).tw,kf.	494
43	((street or streets) adj15 (transition* or transfer*)).tw,kf.	133
44	(community adj15 (transition* or transfer*)).tw,kf.	6579
45	(rehabilitation adj15 (transition* or transfer*)).tw,kf.	2859
46	or/1-45 [Care Transitions]	430076
47	Patient Navigation/	1022
48	patient navigation.tw,kf.	1092
49	navigator*.tw,kf.	4289
50	care coordinator*.tw,kf.	900
51	"care co ordinator*".tw,kf.	52
52	Case Managers/	275
53	case manager*.tw,kf.	3896
54	peer group/ or peer influence/	24405
55	counseling/	39263
56	social support/ or psychosocial support systems/	78896
57	54 and (55 or 56)	3655
58	(peer health mentor* or peer health coach* or peer coach* or peer mentor* or peer navigation or peer recovery coach* or peer support* or "peer to peer" or peer worker*).tw,kf.	10060
59	transition* coach*.tw,kf.	25
60	(health* coach or health* coaches or health* coaching).tw,kf.	1267
61	(care coach or care coaches or care coaching).tw,kf.	50
62	Social Workers/	1050
63	"social worker*".tw,kf.	11805
64	or/47-53,57-63	35196
65	46 and 64 [Care Transitions and Q1 Navigator]	6173

66	limit 65 to english language	5951
67	limit 66 to ed=20210603-20230117	943
68	("202106*" or "20210629" or "20210630" or "202107*" or "202108*" or "202109*" or "202110*" or "202111*" or "202112*" or "2022*" or "20230101" or "20230101" or "20230102" or "20230103" or "20230104" or "20230105" or "20230106" or "20230107" or "20230108" or "20230109" or "20230110" or "20230111" or "20230112" or "20230113" or "20230114" or "20230115" or "20230116" or "20230117" or "20230118").dt.	2609394
69	66 and 68	918
70	69 not ("20210601" or "20210602").dt.	913
71	67 or 70	1260

Database: Cumulative Index to Nursing and Allied Health (CINAHL)
Search strategy:

#	Query	Last Run Via	Results
S1	(MH "Continuity of Patient Care")	Interface - EBSCOhost Research Databases<br>Search Screen - Advanced Search<br>Database - CINAHL Ultimate	16,981
S2	TI (interfacilit* N15 (coordinat* OR "co ordinat*")) OR AB (interfacilit* N15 (coordinat* OR "co ordinat*"))	Interface - EBSCOhost Research Databases<br>Search Screen - Advanced Search<br>Database - CINAHL Ultimate	7
S3	TI (inter facilit* N15 (coordinat* OR "co ordinat*")) OR AB (inter facilit* N15 (coordinat* OR "co ordinat*"))	Interface - EBSCOhost Research Databases<br>Search Screen - Advanced Search<br>Database - CINAHL Ultimate	6
S4	TI (intrafacilit* N15 (coordinat* OR "co ordinat*")) OR AB (intra facilit* N15 (coordinat* OR "co ordinat*"))	Interface - EBSCOhost Research Databases<br>Search Screen - Advanced Search<br>Database - CINAHL Ultimate	0
S5	TI (intra facilit* N15 (coordinat* OR "co ordinat*")) OR AB (intra facilit* N15 (coordinat* OR "co ordinat*"))	Interface - EBSCOhost Research Databases<br>Search Screen - Advanced Search<br>Database - CINAHL Ultimate	0
S6	TI (care N5 (continuum* OR continuit*)) OR AB (care N5 (continuum* OR continuit*))	Interface - EBSCOhost Research Databases<br>Search Screen - Advanced Search<br>Database - CINAHL Ultimate	12,739
S7	TI (care N5 (coordinat* OR "co ordinat*")) OR AB (care N5 (coordinat* OR "co ordinat*"))	Interface - EBSCOhost Research Databases<br>Search Screen - Advanced Search<br>Database - CINAHL Ultimate	12,848

S8	TI (healthcare N5 (continuum* OR continuit*)) OR AB (healthcare N5 (continuum* OR continuit*))	Interface - EBSCOhost Research Databases<br>Search Screen - Advanced Search<br>Database - CINAHL Ultimate	1,328
S9	TI (interfacilt* N5 (continuum* OR continuit*)) OR AB (interfacilt* N5 (continuum* OR continuit*))	Interface - EBSCOhost Research Databases<br>Search Screen - Advanced Search<br>Database - CINAHL Ultimate	0
S10	TI (inter facilt* N5 (continuum* OR continuit*)) OR AB (inter facilt* N5 (continuum* OR continuit*))	Interface - EBSCOhost Research Databases<br>Search Screen - Advanced Search<br>Database - CINAHL Ultimate	0
S11	TI (intrafacilt* N5 (continuum* OR continuit*)) OR AB (intrafacilt* N5 (continuum* OR continuit*))	Interface - EBSCOhost Research Databases<br>Search Screen - Advanced Search<br>Database - CINAHL Ultimate	0
S12	TI (intra facilt* N5 (continuum* OR continuit*)) OR AB (intra facilt* N5 (continuum* OR continuit*))	Interface - EBSCOhost Research Databases<br>Search Screen - Advanced Search<br>Database - CINAHL Ultimate	0
S13	TI (healthcare N5 (coordinat* OR "co ordinat*")) OR AB (healthcare N5 (coordinat* OR "co ordinat*"))	Interface - EBSCOhost Research Databases<br>Search Screen - Advanced Search<br>Database - CINAHL Ultimate	2,111
S14	TI (healthcare N5 (continuum* OR continuit*)) OR AB (healthcare N5 (continuum* OR continuit*))	Interface - EBSCOhost Research Databases<br>Search Screen - Advanced Search<br>Database - CINAHL Ultimate	1,328
S15	(MH "Health Care Delivery")	Interface - EBSCOhost Research Databases<br>Search Screen - Advanced Search<br>Database - CINAHL Ultimate	64,492
S16	TI "delivery of health care" OR AB "delivery of health care"	Interface - EBSCOhost Research Databases<br>Search Screen - Advanced Search<br>Database - CINAHL Ultimate	1,125
S17	TI "delivery of healthcare" OR AB "delivery of healthcare"	Interface - EBSCOhost Research Databases<br>Search Screen - Advanced Search<br>Database - CINAHL Ultimate	600
S18	TI "health care delivery" OR AB "health care delivery"	Interface - EBSCOhost Research Databases<br>Search Screen - Advanced Search<br>Database - CINAHL Ultimate	6,196

S19	TI "healthcare delivery" OR AB "healthcare delivery"	Interface - EBSCOhost Research Databases<br>Search Screen - Advanced Search<br>Database - CINAHL Ultimate	3,804
S20	(MH "Health Care Delivery, Integrated")	Interface - EBSCOhost Research Databases<br>Search Screen - Advanced Search<br>Database - CINAHL Ultimate	14,645
S21	TI (integrat* N3 (care OR healthcare)) OR AB (integrat* N3 (care OR healthcare))	Interface - EBSCOhost Research Databases<br>Search Screen - Advanced Search<br>Database - CINAHL Ultimate	22,189
S22	TI (site# N2 care) OR AB (site# N2 care)	Interface - EBSCOhost Research Databases<br>Search Screen - Advanced Search<br>Database - CINAHL Ultimate	2,967
S23	(MH "Early Patient Discharge") OR (MH "Patient Discharge")	Interface - EBSCOhost Research Databases<br>Search Screen - Advanced Search<br>Database - CINAHL Ultimate	23,978
S24	TI (patient N15 discharg*) OR AB (patient N15 discharg*)	Interface - EBSCOhost Research Databases<br>Search Screen - Advanced Search<br>Database - CINAHL Ultimate	48,951
S25	TI (hospital N15 discharge*) OR AB (hospital N15 discharge*)	Interface - EBSCOhost Research Databases<br>Search Screen - Advanced Search<br>Database - CINAHL Ultimate	34,874
S26	TI (discharg* N15 (home or homes)) OR AB (discharg* N15 (home or homes))	Interface - EBSCOhost Research Databases<br>Search Screen - Advanced Search<br>Database - CINAHL Ultimate	10,814
S27	(MH "Discharge Planning")	Interface - EBSCOhost Research Databases<br>Search Screen - Advanced Search<br>Database - CINAHL Ultimate	5,601
S28	TI (discharg* N15 plan*) OR AB (discharg* N15 plan*)	Interface - EBSCOhost Research Databases<br>Search Screen - Advanced Search<br>Database - CINAHL Ultimate	5,534
S29	(MH "Transfer, Discharge")	Interface - EBSCOhost Research Databases<br>Search Screen - Advanced Search<br>Database - CINAHL Ultimate	6,617

S30	TI (patient* N15 (transition* OR transfer*)) OR AB (patient* N15 (transition* OR transfer*))	Interface - EBSCOhost Research Databases<br>Search Screen - Advanced Search<br>Database - CINAHL Ultimate	25,261
S31	(MH "Transitional Care")	Interface - EBSCOhost Research Databases<br>Search Screen - Advanced Search<br>Database - CINAHL Ultimate	3,374
S32	TI (care N15 (transition* or transfer*)) OR AB (care N15 (transition* or transfer*))	Interface - EBSCOhost Research Databases<br>Search Screen - Advanced Search<br>Database - CINAHL Ultimate	21,617
S33	TI (healthcare N15 (transfer* or transition*)) OR AB (healthcare N15 (transfer* or transition*))	Interface - EBSCOhost Research Databases<br>Search Screen - Advanced Search<br>Database - CINAHL Ultimate	5,879
S34	TI (facility N15 (transfer* OR transition*)) OR AB (facility N15 (transfer* OR transition*))	Interface - EBSCOhost Research Databases<br>Search Screen - Advanced Search<br>Database - CINAHL Ultimate	2,303
S35	TI (facilities N15 (transfer* OR transition*)) OR AB (facilities N15 (transfer* OR transition*))	Interface - EBSCOhost Research Databases<br>Search Screen - Advanced Search<br>Database - CINAHL Ultimate	2,303
S36	TI ("patient turfing*") OR AB ("patient turfing*")	Interface - EBSCOhost Research Databases<br>Search Screen - Advanced Search<br>Database - CINAHL Ultimate	2
S37	TI (home N15 (transition* or transfer*)) OR AB (home N15 (transition* or transfer*))	Interface - EBSCOhost Research Databases<br>Search Screen - Advanced Search<br>Database - CINAHL Ultimate	4,708
S38	TI (homes N15 (transition* or transfer*)) OR AB (homes N15 (transition* or transfer*))	Interface - EBSCOhost Research Databases<br>Search Screen - Advanced Search<br>Database - CINAHL Ultimate	4,708
S39	TI (floor N15 (transition* or transfer*)) OR AB (floor N15 (transition* or transfer*))	Interface - EBSCOhost Research Databases<br>Search Screen - Advanced Search<br>Database - CINAHL Ultimate	211
S40	TI (setting* N15 (transition* or transfer*)) OR AB (setting* N15 (transition* or transfer*))	Interface - EBSCOhost Research Databases<br>Search Screen - Advanced Search<br>Database - CINAHL Ultimate	4,452

S41	(MH "Transfer, Intrahospital")	Interface - EBSCOhost Research Databases<br>Search Screen - Advanced Search<br>Database - CINAHL Ultimate	1,333
S42	TI ((hospital or hospitals or intrahospital* or intrafacilit* or interfacilit* or interhospital*) N15 transfer*) OR AB ((hospital or hospitals or intrahospital* or intrafacilit* or interfacilit* or interhospital*) N15 transfer*)	Interface - EBSCOhost Research Databases<br>Search Screen - Advanced Search<br>Database - CINAHL Ultimate	6,461
S43	TI ((hospital or hospitals or intrahospital* or intrafacilit* or interfacilit* or interhospital*) N15 transition*) OR AB ((hospital or hospitals or intrahospital* or intrafacilit* or interfacilit* or interhospital*) N15 transition*)	Interface - EBSCOhost Research Databases<br>Search Screen - Advanced Search<br>Database - CINAHL Ultimate	3,921
S44	TI ((shelter or shelters) N15 (transition* or transfer*)) OR AB ((shelter or shelters) N15 (transition* or transfer*))	Interface - EBSCOhost Research Databases<br>Search Screen - Advanced Search<br>Database - CINAHL Ultimate	118
S45	TI ((hospice or hospices) N15 (transition* or transfer*)) OR AB ((hospice or hospices) N15 (transition* or transfer*))	Interface - EBSCOhost Research Databases<br>Search Screen - Advanced Search<br>Database - CINAHL Ultimate	402
S46	TI ((street or streets) N15 (transition* or transfer*)) OR AB ((street or streets) N15 (transition* or transfer*))	Interface - EBSCOhost Research Databases<br>Search Screen - Advanced Search<br>Database - CINAHL Ultimate	72
S47	TI (community N15 (transition* or transfer*)) OR AB (community N15 (transition* or transfer*))	Interface - EBSCOhost Research Databases<br>Search Screen - Advanced Search<br>Database - CINAHL Ultimate	4,167
S48	TI (rehabilitation N15 (transition* or transfer*)) OR AB (rehabilitation N15 (transition* or transfer*))	Interface - EBSCOhost Research Databases<br>Search Screen - Advanced Search<br>Database - CINAHL Ultimate	1,831
S49	TI (system N5 navigat*) OR AB (system N5 navigat*)	Interface - EBSCOhost Research Databases<br>Search Screen - Advanced Search<br>Database - CINAHL Ultimate	3,292
S50	(MH "Hospital to Home Transition")	Interface - EBSCOhost Research Databases<br>Search Screen - Advanced Search<br>Database - CINAHL Ultimate	89

S51	TI ("hospital to home" or "hospital to homes" or "H2H transition*") OR AB ("hospital to home" or "hospital to homes" or "H2H transition*")	Interface - EBSCOhost Research Databases<br>Search Screen - Advanced Search<br>Database - CINAHL Ultimate	1,073
S52	S1 OR S2 OR S3 OR S4 OR S5 OR S6 OR S7 OR S8 OR S9 OR S10 OR S11 OR S12 OR S13 OR S14 OR S15 OR S16 OR S17 OR S18 OR S19 OR S20 OR S21 OR S22 OR S23 OR S24 OR S25 OR S26 OR S27 OR S28 OR S29 OR S30 OR S31 OR S32 OR S33 OR S34 OR S35 OR S36 OR S37 OR S38 OR S39 OR S40 OR S41 OR S42 OR S43 OR S44 OR S45 OR S46 OR S47 OR S48 OR S49 OR S50 OR S51	Interface - EBSCOhost Research Databases<br>Search Screen - Advanced Search<br>Database - CINAHL Ultimate	255,230
S53	MH "Patient Navigation")	Interface - EBSCOhost Research Databases<br>Search Screen - Advanced Search<br>Database - CINAHL Ultimate	1,907
S54	TI ("patient navigation") OR AB ("patient navigation")	Interface - EBSCOhost Research Databases<br>Search Screen - Advanced Search<br>Database - CINAHL Ultimate	788
S55	TI navigator* OR AB navigator*	Interface - EBSCOhost Research Databases<br>Search Screen - Advanced Search<br>Database - CINAHL Ultimate	2,528
S56	TI "care coordinator*" OR AB "care coordinator*"	Interface - EBSCOhost Research Databases<br>Search Screen - Advanced Search<br>Database - CINAHL Ultimate	846
S57	TI ("care co ordinator*") OR AB ("care co ordinator*")	Interface - EBSCOhost Research Databases<br>Search Screen - Advanced Search<br>Database - CINAHL Ultimate	68
S58	(MH "Case Managers")	Interface - EBSCOhost Research Databases<br>Search Screen - Advanced Search<br>Database - CINAHL Ultimate	4,464
S59	TI "case manager*" OR AB "case manager*"	Interface - EBSCOhost Research Databases<br>Search Screen - Advanced Search<br>Database - CINAHL Ultimate	4,798
S60	(MH "Peer Group")	Interface - EBSCOhost Research Databases<br>Search Screen - Advanced Search<br>Database - CINAHL Ultimate	16,110

S61	(MH "Counseling")	Interface - EBSCOhost Research Databases<br>Search Screen - Advanced Search<br>Database - CINAHL Ultimate	35,189
S62	(MH "Support, Psychosocial")	Interface - EBSCOhost Research Databases<br>Search Screen - Advanced Search<br>Database - CINAHL Ultimate	96,627
S63	S60 AND (S61 OR S62)	Interface - EBSCOhost Research Databases<br>Search Screen - Advanced Search<br>Database - CINAHL Ultimate	3,422
S64	(MH "Peer Assistance Programs")	Interface - EBSCOhost Research Databases<br>Search Screen - Advanced Search<br>Database - CINAHL Ultimate	1,039
S65	TI ("peer health mentor*" or "peer health coach*" or "peer coach*" or "peer mentor*" or "peer navigation" or "peer recovery coach*" or "peer support*" or "peer to peer" or "peer worker*") OR AB ("peer health mentor*" or "peer health coach*" or "peer coach*" or "peer mentor*" or "peer navigation" or "peer recovery coach*" or "peer support*" or "peer to peer" or "peer worker*")	Interface - EBSCOhost Research Databases<br>Search Screen - Advanced Search<br>Database - CINAHL Ultimate	7,429
S66	TI "transition* coach*" OR AB "transition* coach*"	Interface - EBSCOhost Research Databases<br>Search Screen - Advanced Search<br>Database - CINAHL Ultimate	30
S67	TI ("health* coach" or "health* coaches" or "health* coaching") OR AB ("health* coach" or "health* coaches" or "health* coaching")	Interface - EBSCOhost Research Databases<br>Search Screen - Advanced Search<br>Database - CINAHL Ultimate	937
S68	TI ("care coach" or "care coaches" or "care coaching") OR AB ("care coach" or "care coaches" or "care coaching")	Interface - EBSCOhost Research Databases<br>Search Screen - Advanced Search<br>Database - CINAHL Ultimate	43
S69	(MH "Social Workers")	Interface - EBSCOhost Research Databases<br>Search Screen - Advanced Search<br>Database - CINAHL Ultimate	11,149
S70	TI "social worker*" OR AB "social worker*"	Interface - EBSCOhost Research Databases<br>Search Screen - Advanced Search<br>Database - CINAHL Ultimate	14,187

S71	S53 OR S54 OR S55 OR S56 OR S57 OR S58 OR S59 OR S63 OR S64 OR S65 OR S66 OR S67 OR S68 OR S69 OR S70	Interface - EBSCOhost Research Databases<br>Search Screen - Advanced Search<br>Database - CINAHL Ultimate	43,122
S72	S52 AND S71	Interface - EBSCOhost Research Databases<br>Search Screen - Advanced Search<br>Database - CINAHL Ultimate	6,885
S73	S52 AND S71	Interface - EBSCOhost Research Databases<br>Search Screen - Advanced Search<br>Database - CINAHL Ultimate	6,781
S74	S52 AND S71	Interface - EBSCOhost Research Databases<br>Search Screen - Advanced Search<br>Database - CINAHL Ultimate	5,342
S75	EM 202106*-	Interface - EBSCOhost Research Databases<br>Search Screen - Advanced Search<br>Database - CINAHL Ultimate	549,362
S76	S74 AND S75	Interface - EBSCOhost Research Databases<br>Search Screen - Advanced Search<br>Database - CINAHL Ultimate	777
S38	TI (homes N15 (transition* or transfer*)) OR AB (homes N15 (transition* or transfer*))	Expanders - Apply equivalent subjects<br>Search modes - Boolean/Phrase	4,715
S39	TI (floor N15 (transition* or transfer*)) OR AB (floor N15 (transition* or transfer*))	Expanders - Apply equivalent subjects<br>Search modes - Boolean/Phrase	212
S40	TI (setting* N15 (transition* or transfer*)) OR AB (setting* N15 (transition* or transfer*))	Expanders - Apply equivalent subjects<br>Search modes - Boolean/Phrase	4,463
S41	(MH "Transfer, Intrahospital")	Expanders - Apply equivalent subjects<br>Search modes - Boolean/Phrase	1,333
S42	TI ((hospital or hospitals or intrahospital* or intrafacilit* or interfacilit* or interhospital*) N15 transfer*) OR AB ((hospital or hospitals or intrahospital* or intrafacilit* or interfacilit* or interhospital*) N15 transfer*)	Expanders - Apply equivalent subjects<br>Search modes - Boolean/Phrase	6,472
S43	TI ((hospital or hospitals or intrahospital* or intrafacilit* or interfacilit* or interhospital*) N15 transition*) OR AB ((hospital or hospitals or intrahospital* or	Expanders - Apply equivalent subjects<br>Search modes - Boolean/Phrase	3,929

	intrafacilit* or interfacilit* or interhospital*) N15 transition*)		
S44	TI ((shelter or shelters) N15 (transition* or transfer*)) OR AB ((shelter or shelters) N15 (transition* or transfer*))	Expanders - Apply equivalent subjects Search modes - Boolean/Phrase	118
S45	TI ((hospice or hospices) N15 (transition* or transfer*)) OR AB ((hospice or hospices) N15 (transition* or transfer*))	Expanders - Apply equivalent subjects Search modes - Boolean/Phrase	402
S46	TI ((street or streets) N15 (transition* or transfer*)) OR AB ((street or streets) N15 (transition* or transfer*))	Expanders - Apply equivalent subjects Search modes - Boolean/Phrase	72
S47	TI (community N15 (transition* or transfer*)) OR AB (community N15 (transition* or transfer*))	Expanders - Apply equivalent subjects Search modes - Boolean/Phrase	4,171
S48	TI (rehabilitation N15 (transition* or transfer*)) OR AB (rehabilitation N15 (transition* or transfer*))	Expanders - Apply equivalent subjects Search modes - Boolean/Phrase	1,834
S49	TI (system N5 navigat*) OR AB (system N5 navigat*)	Expanders - Apply equivalent subjects Search modes - Boolean/Phrase	3,295
S50	S1 OR S2 OR S3 OR S4 OR S5 OR S6 OR S7 OR S8 OR S9 OR S10 OR S11 OR S12 OR S13 OR S14 OR S15 OR S16 OR S17 OR S18 OR S19 OR S20 OR S21 OR S22 OR S23 OR S24 OR S25 OR S26 OR S27 OR S28 OR S29 OR S30 OR S31 OR S32 OR S33 OR S34 OR S35 OR S36 OR S38 OR S39 OR S40 OR S41 OR S42 OR S43 OR S44 OR S45 OR S46 OR S47 OR S48 OR S49	Expanders - Apply equivalent subjects Search modes - Boolean/Phrase	255,551
S51	(MH "Cooperative Behavior")	Expanders - Apply equivalent subjects Search modes - Boolean/Phrase	9,060
S52	TI (cooperat* or "co operativ*") OR AB (cooperat* or "co operativ*")	Expanders - Apply equivalent subjects Search modes - Boolean/Phrase	24,046
S53	(MH "Teamwork")	Expanders - Apply equivalent subjects Search modes - Boolean/Phrase	18,990
S54	TI ("team work" or teamwork) OR AB ("team work" or teamwork)	Expanders - Apply equivalent subjects Search modes - Boolean/Phrase	10,030
S55	(MH "Communication") OR (MH "Email") OR (MH "Telecommunications") OR (MH "Instant Messaging") OR (MH "Teleconferencing") OR (MH "Telemedicine") OR (MH "Telehealth") OR (MH "Remote Consultation") OR (MH	Expanders - Apply equivalent subjects Search modes - Boolean/Phrase	162,352

	"Telerehabilitation") OR (MH "Telenursing") OR (MH "Telephone") OR (MH "Cellular Phone") OR (MH "Text Messaging") OR (MH "Smartphone") OR (MH "Videoconferencing")		
S56	TI (communicat* or telecommunication* or electronic mail or email or "e-mail" or remote consult* or teleradiology or telerehab* or telephone* or phone* or smartphone* or text messag* or texting or videoconferenc* or video conferenc*)	Expanders - Apply equivalent subjects Search modes - Boolean/Phrase	61,783
S57	AB (communicat* or telecommunication* or electronic mail or email or "e-mail" or remote consult* or teleradiology or telerehab* or telephone* or phone* or smartphone* or text messag* or texting or videoconferenc* or video conferenc*)	Expanders - Apply equivalent subjects Search modes - Boolean/Phrase	196,656
S58	S51 OR S52 OR S53 OR S54 OR S55 OR S56 OR S57	Expanders - Apply equivalent subjects Search modes - Boolean/Phrase	365,355
S59	(MH "Health Personnel")	Expanders - Apply equivalent subjects Search modes - Boolean/Phrase	51,635
S60	TI ((healthcare or health or medical or nursing) N2 (personnel or practitioner* or professional* or provider* or staff or worker*)) OR AB ((healthcare or health or medical or nursing) N2 (personnel or practitioner* or professional* or provider* or staff or worker*))	Expanders - Apply equivalent subjects Search modes - Boolean/Phrase	244,504
S61	(MH "Multidisciplinary Care Team") OR (MH "Team Nursing")	Expanders - Apply equivalent subjects Search modes - Boolean/Phrase	51,973
S62	TI ((care or health or healthcare or inter profession* or interprofession* or medical or multi disciplinary or multidisciplinary or nursing or trans disciplinary or transdisciplinary) N3 (team or teams)) OR AB ((care or health or healthcare or inter profession* or interprofession* or medical or multi disciplinary or multidisciplinary or nursing or trans disciplinary or transdisciplinary) N3 (team or teams))	Expanders - Apply equivalent subjects Search modes - Boolean/Phrase	48,795
S63	S59 OR S60 OR S61 OR S62	Expanders - Apply equivalent subjects Search modes - Boolean/Phrase	345,884
S64	S58 AND S63	Expanders - Apply equivalent subjects Search modes - Boolean/Phrase	58,130

S65	(MH "Nurse-Physician Relations") OR (MH "Interprofessional Relations")	Expanders - Apply equivalent subjects<br>Search modes - Boolean/Phrase	34,847
S66	TI ((relation* or communicat* or connect* or cooperat* or "co operat*") N2 (interprofession* or professional*)) OR AB ((relation* or communicat* or connect* or cooperat* or "co operat*") N2 (interprofession* or professional*))	Expanders - Apply equivalent subjects<br>Search modes - Boolean/Phrase	9,137
S67	TI ((relation* or communicat* or connect* or cooperat* or "co operat*") N2 (team or teams)) OR AB ((relation* or communicat* or connect* or cooperat* or "co operat*") N2 (team or teams))	Expanders - Apply equivalent subjects<br>Search modes - Boolean/Phrase	3,563
S68	TI ((relation* or communicat* or connect* or cooperat* or "co operat*") N2 (intersector* or inter sector*)) OR AB ((relation* or communicat* or connect* or cooperat* or "co operat*") N2 (intersector* or inter sector*))	Expanders - Apply equivalent subjects<br>Search modes - Boolean/Phrase	108
S69	TI ((relation* or communicat* or connect* or cooperat* or "co operat*") N2 (crossdisciplinary or cross disciplinary or interdisciplinary communication* or inter disciplinary or multidisciplinary or multi disciplinary or trans disciplinary or transdisciplinary)) OR AB ((relation* or communicat* or connect* or cooperat* or "co operat*") N2 (crossdisciplinary or cross disciplinary or interdisciplinary communication* or inter disciplinary or multidisciplinary or multi disciplinary or trans disciplinary or transdisciplinary))	Expanders - Apply equivalent subjects<br>Search modes - Boolean/Phrase	1,236
S70	TI ((doctor* or physician* or clinician* or surgeon*) N3 (nurse or nurses) N2 relation*) OR AB ((doctor* or physician* or clinician* or surgeon*) adj3 (nurse or nurses) N2 relation*)	Expanders - Apply equivalent subjects<br>Search modes - Boolean/Phrase	239
S71	TI ((doctor* or physician* or clinician* or surgeon*) N3 (nurse or nurses) N2 communicat*) OR AB ((doctor* or physician* or clinician* or surgeon*) N3 (nurse or nurses) N2 communicat*)	Expanders - Apply equivalent subjects<br>Search modes - Boolean/Phrase	548
S72	TI ((doctor* or physician* or clinician* or surgeon*) N3 (nurse or nurses) N2 collaborat*) OR AB ((doctor* or	Expanders - Apply equivalent subjects<br>Search modes - Boolean/Phrase	789

	physician* or clinician* or surgeon*) N3 (nurse or nurses) N2 collaborat*)		
S73	TI ((doctor* or physician* or clinician* or surgeon*) N3 (nurse or nurses) N2 (cooperat* or "co operat*")) OR AB ((doctor* or physician* or clinician* or surgeon*) N3 (nurse or nurses) N2 (cooperat* or "co operat*"))	Expanders - Apply equivalent subjects<br>Search modes - Boolean/Phrase	81
S74	TI ((doctor* or physician* or clinician* or surgeon*) N3 (nurse or nurses) N2 connect*) OR AB ((doctor* or physician* or clinician* or surgeon*) N3 (nurse or nurses) N2 connect*)	Expanders - Apply equivalent subjects<br>Search modes - Boolean/Phrase	15
S75	S64 OR S65 OR S66 OR S67 OR S68 OR S69 OR S70 OR S71 OR S72 OR S73 OR S74	Expanders - Apply equivalent subjects<br>Search modes - Boolean/Phrase	95,767
S76	S50 AND S75	Expanders - Apply equivalent subjects<br>Search modes - Boolean/Phrase	15,363
S77	S50 AND S75	Limiters - English Language; Peer Reviewed<br>Expanders - Apply equivalent subjects<br>Search modes - Boolean/Phrase	13,383
S78	EM 20210628*-	Limiters - English Language; Peer Reviewed<br>Expanders - Apply equivalent subjects<br>Search modes - Boolean/Phrase	511,179
S79	S77 AND S78	Expanders - Apply equivalent subjects<br>Search modes - Boolean/Phrase	1,866
