# Supplementary material for: Navigation Support during Transitions in Care for Persons with Complex Care Needs: A Systematic Review
Source: Healthcare (Basel). 2024 Sep 10;12(18):1814. doi: 10.3390/healthcare12181814 (PMC11431248; doi:10.3390/healthcare12181814)
Supplement: Supplementary file 1 [file healthcare-12-01814-s001.zip › File S2_Evidence profile.pdf]

Evidence Profile

Question: Should support from a system navigator be recommended or not for persons encountering a transition in care?

Population: Adult & pediatric populations with complex care needs experiencing a transition in care
Intervention: Support from a system navigator
Comparison: No support from a system navigator
Outcome: Patient quality of life (QOL), emergency department (ED) visits (within 30 days of a transition in care), follow-up visit with a health or social service provider, patient satisfaction, readmission rates (within 30 days of a transition in care)

Setting: Any setting where a person receives care or services during a transition in care

Quality assessment							Reference	Study details		No. of Participants		Reported effects/outcomes	Certainty
№ of studies	Study design	Risk of bias	Inconsistency	Indirectness	Imprecision	Publication bias		Country	Intervention	Intervention	Control		
**Patient QOL** *(measured using the EQ-5D-3L, SGRQ, PROMIS Global-10, MQOL-HK, CHQ-C and SWL questionnaires)*													
3	RCT	Serious [a]	Not serious [b]	Not serious [c]	Not serious [d]	Undetected						The studies showed no important differences in QOL between groups when reviewing the size of effects.	⊕◯◯◯ Very low
							Rose et al., 2018[1]	Canada	**Intervention:** In addition to usual care, the intervention group received support from a case manager after discharged home from hospital. The case manager provided education about COPD, individualized action plans to recognize and manage COPD exacerbations, telephone consultations, and motivational interviewing focusing on health behaviours.<br><br>**Control:** Usual care consisted of 3 monthly outpatient clinic visits,	N=236<br><br>Mean (SD)<br><br>Generic Health related QOL (EQ-5D-3L):<br><br>Baseline: 58 (20)<br>3 months: 63 (19)<br>6 months: 62 (18)<br>12 months: 65 (19)<br><br>Disease specific health related QOL (SGRQ):	N=234<br><br>Mean (SD)<br><br>Generic Health related QOL (EQ-5D-3L):<br><br>Baseline: 60 (17)<br>3 months: 61 (17)<br>6 months: 62 (18)<br>12 months: 63 (17)<br><br>Disease specific health related QOL (SGRQ:)	Post intervention, there were no important differences in generic or disease specific QOL between the intervention and control group.<br><br>.	

Evidence Profile

Quality assessment							Reference	Study details		No. of Participants		Reported effects/outcomes	Certainty
№ of studies	Study design	Risk of bias	Inconsistency	Indirectness	Imprecision	Publication bias		Country	Intervention	Intervention	Control		
									an 8-week rehab program, and an individualized action plan.	Baseline: 53 (19)<br>3 months: 48 (19)<br>6 months: 48 (19)<br>12 months: 48 (21)	Baseline: 54 (20)<br>3 months: 54 (20)<br>6 months: 51 (20)<br>12 months: 52 (21)		
							Reeves et al., 2019[2]	USA	**Intervention:** Persons with stroke transitioning from hospital to home received a multi-component social worker-led case management intervention that included development of a service plan to address person's goals and priorities, arranging appointments and community service referrals, and offering practical and emotional support.<br><br>**Control:** Usual care consisted of standard post-discharge instructions and services including med lists, education materials, follow-up instructions and referrals.	N=88<br><br>Least Square Mean [95% CI]<br><br>PROMIS -10 Global Physical Health<br><br>7 days: 41.9 [40.6 to 43.3]<br>90 days: 43.2 [41.8 to 44.6]<br><br>PROMIS -10 Global Mental Health<br><br>7 days: 45.2 [43.4 to 47.1]<br>90 days: 45.4 [43.4 to 47.3]	N=87<br><br>Least Square Mean [95% CI]<br><br>PROMIS -10 Global Physical Health<br><br>7 days: 42.8 [41.4 to 44.14]<br>90 days: 43.1 [41.7 to 44.5]<br><br>PROMIS -10 Global Mental Health<br><br>7 days: 46.0 [44.0 to 47.9]<br>90 days: 47.1 [45.2 to 49.1]	Post intervention, there were no important differences in physical or mental health QOL between the intervention and control group.	
							Ng & Wong, 2018[3]	Hong Kong, China	**Intervention:** In addition to usual care, the intervention group	N=41	N=43	Post intervention, QOL was greater in the intervention group than the	

Evidence Profile

Quality assessment							Reference	Study details		No. of Participants		Reported effects/outcomes	Certainty
№ of studies	Study design	Risk of bias	Inconsistency	Indirectness	Imprecision	Publication bias		Country	Intervention	Intervention	Control		
									received support from a palliative nurse case-manager following their transition from hospital to home. As part of the multicomponent intervention, the nurse provided social and spiritual support, helped persons discuss their goals and identify treatment preferences, and made referrals to other providers and health services as necessary.<br><br>**Control:** Usual care involved a predischarge referral to palliative care, standard discharge planning, and a scheduled outpatient appointment. The comparison group also received two social calls.	MQOL-HK – Total Score Mean (95% CI)<br><br>At baseline 6.59 (6.14-7.04)<br><br>After 4 weeks 7.54 (7.14-7.94)<br><br>After 12 weeks 7.49 (7.15-7.83)<br><br>CHQ-C - Total score Median (25th-75th percentile)<br><br>At baseline 4.64 (3.76-5.04)<br><br>After 4 weeks 5.39 (4.18-6.26)<br><br>After 12 weeks 5.41 (4.52-6.01)	MQOL-HK-Total Score Mean (95% CI)<br><br>At baseline 6.37 (5.91-6.83)<br><br>After 4 weeks 6.61 (6.04-7.18)<br><br>After 12 weeks 6.61 (6.17-7.05)<br><br>CHQ-C- Total score Median (25th-75th percentile)<br><br>At baseline 4.55 (3.67-5.40)<br><br>After 4 weeks 4.74 (3.88-5.29)<br><br>After 12 weeks 5.31 (4.69-5.80)	control group over time when MQOL-HK scores were compared.<br><br>At 4 weeks, CHQ-C scores were greater in the intervention group compared to the control group. These differences were not seen at 12 weeks.<br><br>.	
1	Single-arm study	Serious [e]	Not serious	Not serious [c]	Very serious [f]	Undetected	Kidd et al., 2016[4]	Canada	**Intervention**: Peer support workers provided support persons with severe mental illness after discharge from a psychiatric hospital. As part of the multicomponent intervention, peer workers provided	N=31<br><br>Mean (SD) QOL domains at baseline:<br><br>Living situation = 2.52 (1.00)<br>Social relationship = 2.88 (1.00)	No true control group	Post intervention, there was an improvement in self-reported QOL in the domains of 'living situation' with a large effect size and 'social relationships' with a low-medium effect size. There were no differences in the QOL domains of 'self and present life' or 'work'.	⨁◯◯◯<br>Very low

Evidence Profile

Quality assessment							Reference	Study details		No. of Participants		Reported effects/outcomes	Certainty
№ of studies	Study design	Risk of bias	Inconsistency	Indirectness	Imprecision	Publication bias		Country	Intervention	Intervention	Control		
									persons with a 'welcome basket' of needed/desired items and they helped familiarize the person with local resources and supports to facilitate self-management.<br><br>**Control**: There was no control group, and results were compared pre and post intervention.	Work = 2.85 (1.09)<br>Self and present life = 3.02 (0.86)<br><br>Mean (SD) QOL domains post-intervention:<br><br>Living situation = 3.68 (0.80)<br>Social relationship = 3.22 (0.79)<br>Work = 2.82 (1.14)<br>Self and present life = 3.14 (0.79)			
**ED visits (within 30 days of a transition in care)** *(measured using data collected from electronic health records (EMRs))*													
4	RCT	Serious [g]	Not serious [h]	Not serious [c]	Not Serious [i]	Undetected						The studies showed no important differences in ED visits within 30 days between groups when reviewing the size of effects.	⊕⊕◯◯<br>Low
							Balaban et al., 2017[5]	USA	**Intervention:** Patient navigators provided support for persons at high risk of readmission transitioning from hospital to home or skilled nursing facility. As part of the multicomponent intervention, patient navigators provided coaching and addressed barriers to obtaining or taking	N=747<br><br>Events per person in first 30 days post-discharge:<br><br>Persons 60+<br>0.112<br><br>Persons < 60<br>0.644	N=1190<br><br>Events per person in first 30 days post-discharge:<br><br>Persons 60+<br>0.135<br><br>Persons < 60<br>0.358	For persons 60+, there was a slight trend towards a reduction in ED visits within the intervention group compared to the control group in the first 30 days post-discharge.<br><br>For persons <60 years, there was a trend towards an increase in ED visits in the intervention group compared to the control group in the first 30 days post-discharge.	

Evidence Profile

Quality assessment							Reference	Study details		No. of Participants		Reported effects/outcomes	Certainty
**№ of studies**	**Study design**	**Risk of bias**	**Inconsistency**	**Indirectness**	**Imprecision**	**Publication bias**		**Country**	**Intervention**	**Intervention**	**Control**		
									medications, arranged transportation, made referrals for services, facilitated communication with PCP offices, assisted with health insurance problems, and supported patient self-management<br><br>**Control:** The control group received usual inpatient, transitional, and outpatient care.				
							Carter et al., 2021[6]	USA	543: **Intervention:** In the intervention group, community health workers supported persons at high risk for readmission during the transition from hospital to home or a short-stay rehabilitation unit. They provided health coaching, arranged clinical services and social resources, and provided motivational interviewing, goal-setting, and psychosocial support. The intervention group also received usual care.<br><br>**Control:** The control group received usual care, which included routine care from primary care clinics and any outpatient referrals made by	N=288<br><br>31 participants (11.2%) had an ED visit within 30 days	N=285<br><br>46 participants (16.8%) had an ED visit within 30 days	There was a trend towards a reduction in ED visits within 30 days of discharge in the intervention group compared to the control group. For every 100 people who receive the intervention, 5 less people will have an ED visit within 30 days (ranges from 9 less to 0 more, RR 0.67; 95% CI, 0.44-1.02)	

Evidence Profile

Quality assessment							Reference	Study details		No. of Participants		Reported effects/outcomes	Certainty
**№ of studies**	**Study design**	**Risk of bias**	**Inconsistency**	**Indirectness**	**Imprecision**	**Publication bias**		**Country**	**Intervention**	**Intervention**	**Control**		
									hospital case management or social work at the time of discharge.				
							Mitchell et al., 2022[7]	USA	**Intervention**: Participants with moderate to severe depression transitioning from hospital to home received 12-weeks of post-discharge telehealth support from a counselor that included cognitive behavioural therapy, self-management education, and patient navigation. Patient navigation consisted of: support to schedule appointments and transportation, adherence to the discharge plan, and information sharing with primary care clinicians.<br><br>**Control**: The control group received a post-discharge telephone call by a discharge educator who discussed medication adherence, confirmed follow-up appointments, and provided education about symptoms and care plan management.	N= 353<br><br>32 participants (10%) had an ED visit within 30 days	N=356<br><br>32 participants (9%) had an ED visit within 30 days	There were no important differences in ED visits between the intervention and control group at 30 days (10% vs. 9%),<br><br>IRR 1.05; 95% CI 0.64-1.72	

Evidence Profile

Quality assessment							Reference	Study details		No. of Participants		Reported effects/outcomes	Certainty
№ of studies	Study design	Risk of bias	Inconsistency	Indirectness	Imprecision	Publication bias		Country	Intervention	Intervention	Control		
							Taylor et al., 2022[8]	USA	**Intervention**: Participants with sepsis at high risk of readmission, received usual care plus support from a sepsis nurse navigator for 30 days after discharge. The navigator promoted care planning, self-management, follow-up appointments, and patient, provider and community engagement. They also treated health concerns and adjusted medications.<br><br>**Control**: The control group received usual care including patient education and follow-up instructions at discharge, routine recommendations for follow-up visits with primary care provider, and arrangements for home care services.	N= 349<br><br>ED visits in 30 days<br><br>Mean = 0.2 ± 1.1<br>Median = 0 (0-0)	N= 342<br><br>ED visits in 30 days<br><br>Mean= 0.2 ± 0.7<br>Median = 0 (0-0)	There were no important differences in ED visits between the intervention and control group at 30 days<br><br>OR (95% CI) = 1.12 (0.71-1.78)	
**Follow-up visit with a health or social service provider** *(measured using data collected from an EMR, a hospital database, and self-reported primary care visits collected from telephone surveys. Two studies looked at the number of follow-up visits and one study looked at adherence to scheduled follow-up appointments)*													
5	RCT	Serious [j]	Serious [k]	Not serious [c]	Not serious [l]	Undetected						Four studies showed an improvement or trend towards an improvement for follow-up visits with a health or social service provider. One study showed no important differences.	⊕◯◯◯<br>Very low

Evidence Profile

Quality assessment							Reference	Study details		No. of Participants		Reported effects/outcomes	Certainty
№ of studies	Study design	Risk of bias	Inconsistency	Indirectness	Imprecision	Publication bias		Country	Intervention	Intervention	Control		
							Balaban et al., 2017[5]	USA	**Intervention:** Patient navigators provided support for persons at high risk of readmission transitioning from hospital to home or skilled nursing facility. As part of the multicomponent intervention, patient navigators provided coaching and addressed barriers to obtaining or taking medications, arranged transportation, made referrals for services, facilitated communication with PCP offices, assisted with health insurance problems, and supported patient self-management.<br><br>**Control:** The control group received usual inpatient, transitional, and outpatient care.	N=747<br><br>Events per person per 30-day period:<br><br>Persons 60+ Days 1-30: 1.897 Cumulative (over 180 days): 6.942<br><br>Persons < 60 Days 1-30: 2.250 Cumulative (over 180 days):8.918	N=1190<br><br>Events per person per 30-day period:<br><br>Persons 60+ Days 1-30: 1.640 Cumulative (over 180 days): 6.499<br><br>Persons < 60 Days 1-30: 2.008 Cumulative (over 180 days):8.063	For persons 60+, there was an increase in outpatient visits in the first 30 days post-discharge and a trend towards a cumulative increase in outpatient visits 180 days post-discharge in the intervention group compared to the control group.<br><br>For persons <60 years, there was a trend towards an increase in outpatient visits in the first 30 days post-discharge and a trend towards a cumulative increase in outpatient visits 180 days post-discharge in the intervention group compared to the control group.	
							Carter et al., 2021[6]	USA	**Intervention:** In the intervention group, community health workers supported persons at high risk for readmission during the transition from hospital to home or a short-stay rehabilitation unit. They provided health coaching, arranged clinical services and social resources, and provided motivational	N=288<br><br>61 persons [22.0%] had missed an outpatient appointment with primary care or specialist physician within the first 30 days post-discharge	N=285<br><br>92 persons [33.7%] had missed an outpatient appointment with a primary care or specialist physician within the first 30 days post-discharge	There were fewer missed outpatient appointments within 30 days of discharge in the intervention group compared to the control group. For every 100 people who receive the intervention, 11 less people will have a missed outpatient appointment within 30 days of discharge (ranges from 16 less to 4 less; RR 0.66; 95% CI 0.50-0.87)	

Evidence Profile

Quality assessment							Reference	Study details		No. of Participants		Reported effects/outcomes	Certainty
№ of studies	Study design	Risk of bias	Inconsistency	Indirectness	Imprecision	Publication bias		Country	Intervention	Intervention	Control		
									interviewing, goal-setting, and psychosocial support. The intervention group also received usual care.<br><br>**Control:** The control group received usual care, which included routine care from primary care clinics and any outpatient referrals made by hospital case management or social work at the time of discharge.				
							Seaberg et al., 2017[9]	USA	**Intervention:** In the intervention group, patient navigators provided support to persons who had 5 or more ED visits within 12 months to review diagnoses and prescriptions, arrange follow-up appointments and transportation, and identify community resources.<br><br>**Control:** The control group received standard care	N=148<br><br>Average number of follow-up visits at 12 months= 6.42 (95% CI = 5.14–7.70)	N=134<br><br>Average number of follow-up visits at 12 months= 4.07 (95% CI = 3.38–4.76)	At 12 months, there was an increase in follow-up visits in the intervention group compared to the control group.	
							LaBedz et al., 2022[10]	USA	**Intervention:** In the intervention group, hospitalized patients received support from community health workers and peer coaches for 60 days	N=518<br><br>Within 14 days, 232 participants reported an outpatient visit	N= 511<br><br>Within 14 days, 236 participants had an outpatient visit	There were no important differences in outpatient visits within 14 days of discharge in the intervention and control group.	

Evidence Profile

Quality assessment							Reference	Study details		No. of Participants		Reported effects/outcomes	Certainty
№ of studies	Study design	Risk of bias	Inconsistency	Indirectness	Imprecision	Publication bias		Country	Intervention	Intervention	Control		
									post-discharge who identified and addressed barriers to care, promoted self-management, and provided a personalized discharge tool to address follow-up visits and tests and medications and lifestyle changes.<br><br>**Control**: The control group received usual care as per their treating medical team	within 14 days of discharge	within 14 days post discharge	OR (95% CI) = 1.0 (0.78- 1.3)	
							Taylor et al., 2022[8]	USA	**Intervention**: Participants with sepsis at high risk of readmission, received usual care plus support from a sepsis nurse navigator for 30 days after discharge. The navigator promoted care planning, self-management, follow-up appointments, and patient, provider and community engagement. They also treated health concerns and adjusted medications.<br><br>**Control**: The control group received usual care including patient education and follow-up instructions at discharge, routine recommendations for follow-up visits with	N= 349<br><br>136 participants (39%) had an outpatient follow-up visit with a physician within 10 days of hospitalization	N= 342<br><br>107 participants (31%) had an outpatient follow-up visit with a physician within 10 days of hospitalization	Post-intervention there was a trend towards an increase in outpatient follow-up visits in the intervention group compared to the control group. For every 100 people who receive the intervention, 8 more people will have follow-up visit with a physician (ranges from 1 more to 16 more; RR 1.25; 95% CI 1.02-1.53)	

Evidence Profile

Quality assessment							Reference	Study details		No. of Participants		Reported effects/outcomes	Certainty
№ of studies	Study design	Risk of bias	Inconsistency	Indirectness	Imprecision	Publication bias		Country	Intervention	Intervention	Control		
									primary care provider, and arrangements for home care services.				
1	Non-RCT	Serious [m]	Not serious	Not serious [c]	Very serious [n]	Undetected	Pang et al.,2023[11]	Australia	**Intervention:** The intervention group received weekly telephone follow-up support for 30 days post-discharge by a registered nurse (RN) who arranged follow-up appointments and community services, provided appointment reminders, and addressed barriers to care. The RN also sent correspondence to general practitioners to ensure continuum of care.<br><br>**Control**: The control group received usual care including standard medical management processes such as follow-up appointments by the treating ward team.	N=63<br><br>42 participants (66.7%) had a follow-up medical appointment scheduled within 90 days post-discharge	N=262<br><br>96 participants (33.6%) had a follow-up medical appointment scheduled within 90 days post-discharge	Participants who received the intervention had more scheduled medical outpatient appointments within 90 days post-discharge than those in the control group. For every 100 people who receive intervention, 29 more people will have follow-up visit with a health or social service provider (ranges from 15 more to 45 more; RR 1.82; 95% CI 1.44-2.3)	⊕◯◯◯<br>Very low
**Patient satisfaction** *(measured using the CSQ-8, the patient satisfaction questionnaire, phone surveys and a self-developed questionnaire)*													
4	RCT	Serious [o]	Very serious [p]	Not serious [c]	Not serious	Undetected						Overall, two studies showed an increase in satisfaction in the intervention compared to the control, and two studies showed no important differences.	⊕◯◯◯<br>Very Low
							Johnson et al., 2018[12]	UK	**Intervention**: In the intervention group,	N=221	N=220	At 4 months, overall satisfaction with	

Evidence Profile

Quality assessment							Reference	Study details		No. of Participants		Reported effects/outcomes	Certainty
№ of studies	Study design	Risk of bias	Inconsistency	Indirectness	Imprecision	Publication bias		Country	Intervention	Intervention	Control		
									participants discharged from mental health crisis resolution teams received 4 months of support from a peer support worker who supported them in completing a personal recovery workbook which included: setting personal recovery goals, making plans to re-establish a support network, identifying early warning signs, formulating an action plan to avoid relapse and identifying strategies to maintain wellbeing.<br><br>**Control:** The control group received the personal recovery workbook by post and were invited to complete it independently. Participants in both groups also received usual care, with no treatments withheld.	Mean (SD) satisfaction with mental health services 4 months:<br>26 (5)	Mean (SD) satisfaction with mental health services 4 months:<br>24 (6)	mental health-care received was greater in the intervention group than in the control group.	
							Rose et al., 2018[1]	Canada	**Intervention:** In addition to usual care, the intervention group received support from a case manager after discharge from hospital. The case manager provided education about COPD, individualized action plans to recognize and manage	N=236<br><br>Mean (SD)<br><br>1 month:<br>29.5 (3.0)<br>3 months:<br>30.0 (2.8)<br>6 months:<br>29.9 (2.8)<br>12 months:<br>29.9 (2.8)	N=234<br><br>Mean (SD)<br><br>1 month:<br>28.3 (3.7)<br>3 months:<br>28.3 (3.8)<br>6 months:<br>28.0 (3.5)<br>12 months:<br>28.2 (3.6)	Post intervention, there were no important differences in patient satisfaction between the intervention and control group.	

Evidence Profile

Quality assessment							Reference	Study details		No. of Participants		Reported effects/outcomes	Certainty
№ of studies	Study design	Risk of bias	Inconsistency	Indirectness	Imprecision	Publication bias		Country	Intervention	Intervention	Control		
									COPD exacerbations and motivational interviewing focusing on health behaviours.<br><br>**Control:** Usual care consisted of 3 monthly outpatient clinic visits, an 8-week rehab program, and an individualized action plan				
							Ng & Wong, 2018[3]	Hong Kong, China	**Intervention:** In addition to usual care, the intervention group received support from a palliative nurse case-manager following their transition from hospital to home. As part of the multicomponent intervention, the nurse provided social and spiritual support, helped persons discuss their goals and identify treatment preferences, and made referrals to other providers and health services as necessary.<br><br>**Control:** Usual care involved a predischarge referral to palliative care, standard discharge planning, and a scheduled outpatient appointment. The comparison group also received two social calls.	N=41<br><br>Median (25th - 75th percentile)<br><br>After 4 weeks: 4.00 (3.71-4.92)<br><br>After 12 weeks: 4.00 (3.22-4.50)	N=43<br><br>Median (25th - 75th percentile)<br><br>After 4 weeks: 2.92 (2.48-3.92)<br><br>After 12 weeks: 2.76 (2.27-3.77)	At 4 weeks and 12 weeks, satisfaction was greater in the intervention group than in the control group.	

Evidence Profile

Quality assessment							Reference	Study details		No. of Participants		Reported effects/outcomes	Certainty
**№ of studies**	**Study design**	**Risk of bias**	**Inconsistency**	**Indirectness**	**Imprecision**	**Publication bias**		**Country**	**Intervention**	**Intervention**	**Control**		
							Seaberg et al., 2017[9]	USA	**Intervention:** In the intervention group, patient navigators provided to support to persons who had 5 or more ED visits within 12 months to review diagnoses and prescriptions, arrange follow-up appointments and transportation, and identify community resources.<br><br>**Control:** The control group received standard care.	N=148<br><br>Median, 95% CI<br><br>At 2 weeks: 3.00 (95% CI, 2.86 - 3.14)<br><br>At 12 months: 2.00 (95% CI, 1.84 - 2.16)	N=134<br><br>Median, 95% CI<br><br>At 2 weeks: 3.00 (95% CI, 2.87 - 3.13)<br><br>At 12 months: 2.00 (95% CI, 1.84 - 2.16)	Post intervention, there were no important differences in patient satisfaction between the intervention and control group.	
2	Single- arm study	Very serious [q]	Not serious [r]	Not serious [c]	Very serious [s]	Undetected						Overall, both studies reported satisfaction after receiving the intervention.	⊕◯◯◯ Very low
							Samuels et al., 2021[13]	USA	**Intervention:** Patient navigators provided support to frequent ED users after discharge from the ED. As part of the multicomponent intervention, patient navigators helped schedule and remind persons about medical appointments, addressed barriers to care, and made referrals for social needs as needed.<br><br>**Control:** The control group received standard care.	N=49<br><br>All participants reported being overall satisfied with the patient navigator, and 89.7% (35/39) reported being very satisfied.	N=51<br><br>Only the intervention group was surveyed about satisfaction.	Participants reported high overall satisfaction with the patient navigator intervention.	

Evidence Profile

Quality assessment							Reference	Study details		No. of Participants		Reported effects/outcomes	Certainty
№ of studies	Study design	Risk of bias	Inconsistency	Indirectness	Imprecision	Publication bias		Country	Intervention	Intervention	Control		
							Scanlan et al., 2017[14]<br><br>:	Australia	**Intervention:** Following discharge from an inpatient psychiatric unit, persons received 6-8 weeks of support from a peer worker. Supports were tailored to the individual, but were primarily focused on providing practical and emotional support as well as linking participants with community-based supports.<br><br>**Control:** There was no control group.	N=64<br><br>There was a mean rating of 4.4. out of 5 for the following item:<br><br>- Having a support worker with a lived experience has helped me in my recovery	No true control group	Based on a questionnaire filled out at program conclusion, the program appears to be valuable for participants.	
**Readmission rates (within 30 days of a transition in care)** *(measured using data collected from EMRs and an administrative record)*													
5	RCT	Serious [t]	Serious [u]	Not serious [c]	Not serious [v]	Undetected						Three studies reported a decrease or trend towards a decrease in readmission rates within 30 days of a transition in care, one study showed no difference in readmission rates, and one study reported an increase in readmissions for persons over 60 years of age only.	⊕◯◯◯ Very low
							Balaban et al., 2017[5]	USA	**Intervention:** Patient navigators provided support to persons at high risk of readmission transitioning from hospital to home or skilled nursing facility. As part of the multicomponent intervention, patient navigators provided	N=747<br><br>Events per person in first 30 days post-discharge:<br><br>Persons 60+: 0.119<br><br>Persons < 60: 0.312	N=1190<br><br>Events per person in first 30 days post-discharge:<br><br>Persons 60+: 0.147<br><br>Persons < 60: 0.158	For persons 60+, there was a trend towards a decrease in 30-day readmissions in the intervention group compared to the control group.<br><br>For persons < 60, there was an increase in 30-day readmissions in the intervention group	

Evidence Profile

Quality assessment							Reference	Study details		No. of Participants		Reported effects/outcomes	Certainty
№ of studies	Study design	Risk of bias	Inconsistency	Indirectness	Imprecision	Publication bias		Country	Intervention	Intervention	Control		
									coaching and addressed barriers to obtaining or taking medications, arranged transportation, made referrals for services, facilitated communication with other providers, assisted with health insurance problems, and supported patient self-management.<br><br>**<u>Control</u>:** The control group received usual inpatient, transitional, and outpatient care.			compared to the control group.	
							Coller et al., 2018[15]	USA	**<u>Intervention:</u>** In the intervention group, caregivers of children with medical complexity received support from a caregiver coach during the transition from hospital to home. Transition coaches reviewed medication self-management, follow-up appointments, and 'red flags' that could lead to hospitalization and instructions on how to respond to them. They elicited caregiver goals and provided coaching to address the needs identified by families.<br><br>**<u>Control:</u>** The control group received usual care which included	N=77<br><br>17 readmissions per 100 child years	N=70<br><br>23 readmissions per 100 child years	Rates of all-cause 30-day readmissions were reduced in the intervention group compared to the control group (adjusted incident rate ratio: 0.37; 95% CI 0.14–0.98)	

Evidence Profile

Quality assessment							Reference	Study details		No. of Participants		Reported effects/outcomes	Certainty
№ of studies	Study design	Risk of bias	Inconsistency	Indirectness	Imprecision	Publication bias		Country	Intervention	Intervention	Control		
									care planning, case management, and communication with community services.				
							Carter et al., 2021[6]	USA	**Intervention:** In the intervention group, community health workers supported persons at high risk for readmission during the transition from hospital to home or a short-stay rehabilitation unit. They provided health coaching, arranged clinical services and social resources, and provided motivational interviewing, goal-setting, and psychosocial support.<br><br>**Control:** The control group received usual care, which included routine care from primary care clinics and any outpatient referrals made by hospital case management or social work at the time of discharge.	N=288<br><br>35 persons [12.6%] were readmitted within 30 days	N=285<br><br>67 persons [24.5%] were readmitted within 30 days	Compared with participants in the control group, participants in the intervention group were less likely to be readmitted within 30 days. For every 100 people who receive the intervention, 12 less people will be readmitted to hospital within 30 days (ranges from 15 less to 6 less, RR 0.52; 95% CI 0.36-0.75).	
							Mitchell et al., 2022[7]	USA	**Intervention**: Participants with moderate to severe depression transitioning from hospital to home received 12-weeks of post-discharge telehealth support from	N=353<br><br>29 participants (9%) had a readmission within 30 days	N=356<br><br>33 participants (10%) had a readmission within 30 days	There were no important differences in readmissions between the intervention and control group at 30 days (10% vs. 9%; IRR 0.92; 95% CI, 0.56-1.52).	

Evidence Profile

Quality assessment							Reference	Study details		No. of Participants		Reported effects/outcomes	Certainty
№ of studies	Study design	Risk of bias	Inconsistency	Indirectness	Imprecision	Publication bias		Country	Intervention	Intervention	Control		
									a counselor that included cognitive behavioural therapy, self-management education, and patient navigation that included support to schedule appointments and transportation and adhere to the discharge plan, and information sharing with primary care clinicians.<br><br>**Control**: The control received a post-discharge telephone call by a discharge educator who discussed medication adherence, confirmed follow-up appointments, and provided education about symptoms and care plan management.				
							Taylor et al., 2022[8]	USA	**Intervention**: Participants with sepsis at high risk of readmission, received usual care plus support from a sepsis nurse navigator for 30 days after discharge. The navigator promoted care planning, self-management, follow-up appointments, and patient, provider and community engagement. They also treated health	N= 349<br><br>71 participants (20.3%) had a readmission within 30 days	N=342<br><br>84 participants (24.6%) had a readmission within 30d days	Post-intervention there was a decrease in 30-day readmissions in the intervention group compared to the control group.<br><br>OR (95% CI) = 0.78 (0.55-1.12)	

Evidence Profile

Quality assessment							Reference	Study details		No. of Participants		Reported effects/outcomes	Certainty
№ of studies	Study design	Risk of bias	Inconsistency	Indirectness	Imprecision	Publication bias		Country	Intervention	Intervention	Control		
									concerns and adjusted medications.<br><br>**Control**: The control group received usual care including patient education and follow-up instructions at discharge, routine recommendations for follow-up visits with primary care provider, and arrangements for home care services.				
1	Non-RCT	Serious [W]	Not serious	Not serious [c]	Not serious [x]	Undetected	Thompson et al., 2018[16]	USA	**Intervention**: In the intervention group, community navigators supported super utilizers (persons with 11+ hospital encounters in 1 year) during their discharge from hospital. They linked clients to community resources, helped identify and eliminate barriers to health, coordinated care, tailored health information to person's needs, and motivated persons to make healthy choices.<br><br>**Control**: The intervention group was compared to data for super utilizers in contiguous zip codes who did not receive the intervention.	N=159<br><br>30d readmissions - annual average per patient:<br><br>In the year before the intervention was introduced: 2.6<br><br>In the year after the intervention was introduced: 1.6	N=280<br><br>30d readmissions - annual average per patient:<br><br>In the year before the intervention was introduced: 1.6<br><br>In the year after the intervention was introduced: 0.9	There was a trend towards a 18% greater reduction in 30-day readmissions in the intervention group compared to the control group.	⊕◯◯◯<br>Very low

Evidence Profile

Quality assessment							Reference	Study details		No. of Participants		Reported effects/outcomes	Certainty
№ of studies	Study design	Risk of bias	Inconsistency	Indirectness	Imprecision	Publication bias		Country	Intervention	Intervention	Control		
1	Single-arm study	Serious [y]	Not serious	Not serious [c]	Serious [z]	Undetected	Xiang et al., 2019[17]	USA	**Intervention**: Super utilizers (persons with ≥5 hospital admissions in the 12 months), received support from a social worker following a transition from hospital to home. As part of the multicomponent intervention, the social worker helped with care coordination, provided counseling, and arranged services and referrals.<br><br>**Control**: There was no control group, and results were compared pre and post intervention.	N=586<br><br>30-day readmission rate before the intervention (%): 20.78 (18.90, 22.67)<br><br>After the intervention (%): 3.85 (2.79, 4.90)	No true control group	There was a 17% reduction in the 30-day readmission rate 1 month after the intervention.	⊕◯◯◯ Very low

Evidence Profile

Acronyms
CI = Confidence interval
IRR = Incidence rate ratio
OR= Odds ratio
RCT = Randomized controlled trial
RR = Relative risk
SD = Standard deviation

Tools used to measure outcomes

Johnson et al., 2018:
  Client Satisfaction Questionnaire (CSQ-8); higher scores indicate higher satisfaction

Rose et al., 2018:
  EuroQol- 5 Dimension-3 Levels (EQ-5D-3L); scores range from 0-100 and higher scores indicate better QOL
  St. George's Respiratory Questionnaire (SGRQ); scores range from 0-100 and higher scores indicate worse QOL
  Client Satisfaction Questionnaire (CSQ-8); higher scores indicate higher satisfaction

Reeves et al., 2019:
  Patient-Reported Outcomes Measurement Information System Global-10 (PROMIS Global-10); higher scores indicate better QOL

Ng & Wong, 2018:
  McGill Quality of Life Questionnaire-Hong Kong (MQOL-HK); scores range between 0-10 and higher scores indicate better QOL
  The Chronic Heart Failure Questionnaire-Chinese (CHQ-C); 7-point scale and higher scores indicate better QOL
  Patient satisfaction questionnaire; scores range from 1 to 5, higher scores reflect more satisfaction with post discharge care

Samuels et al., 2021:
  Satisfaction was assessed with telephone surveys. Persons were asked how satisfied they were with the intervention (very satisfied, somewhat satisfied, somewhat dissatisfied, very dissatisfied)

Kidd et al., 2016:
  Satisfaction with Life Scale (SWL); higher scores indicate better QOL

Scanlan et al., 2017:
  A self-developed questionnaire; higher scores indicate higher satisfaction

Seaberg et al., 2017:
  Persons were asked about the quality of the service in the ED at 2 weeks and 12 months after the initial ED visit. Satisfaction was measured using a 4-point Likert scale administered via telephone survey

Explanations

a Based on the risk-of bias-tool for randomized trials (RoB 2), there was a serious risk of bias related to the measurement of outcomes and missing data across the three studies. We downgraded by 1.
b The studies showed consistently no difference between groups when reviewing the size of effects. There was inconsistency in the tools used to measure outcomes though. We downgraded by 0.5.
c Although the studies focus on support from a system navigator, the navigator is part of a multicomponent intervention so it is difficult to determine whether the study is truly evaluating the effects of a system navigator providing care. We downgraded by 0.5.
d The total number of participants is less than the optimal 800 participants (n=729). We downgraded by 0.5.
e The study was assessed using the ROBINS-I tool for non-RCT studies, and had serious risk of bias due to lack of control for confounding variables, deviations from the intended intervention, missing data, and self-reporting of outcomes. We downgraded by 1.5
f The total number of participants is far less than the optimal 800 participants (n=31). We downgraded by 2.
g Based on the risk-of bias-tool for randomized trials (RoB 2), there was serious risk of bias related to the randomization process, deviations from the intended interventions, and selection of the reported results across the four studies. We downgraded by 1.
h There were no important differences in ED between groups when reviewing the size of effects. We did not downgrade.
i The number of events is greater than 300. We did not downgrade.
j Based on the risk-of bias-tool for randomized trials (RoB 2), there was very serious risk of bias related to the randomization process, deviations from the intended interventions, missing data, measurement of outcomes and selection of reported results across the five studies. We downgraded by 2.
k One study showed no important differences, but four studies showed an improvement or a trend towards an improvement for this outcome. There was also some inconsistency in the tools used for outcome measurement. We downgraded by 1.
l The number of events is over 300. We did not downgrade.
m The study was assessed using the ROBINS-I tool for non-RCT studies, and had serious risk of bias due to lack of control for confounding variables. We downgraded by 1.
n There were 138 medical follow-up visits. We downgraded by 2.
o Based on the risk-of bias-tool for randomized trials (RoB 2), there was serious risk of bias related to the randomization process, deviations from the intended interventions, missing data, measurement of outcomes and selection of reported results across the four studies. We downgraded by 1.5.
p There was some inconsistency across studies related to tools used for outcome measurement. There was also inconsistency in the reported effects with two studies showing no differences in satisfaction and two studies showing an improvement. We downgraded by 2.
q Based on the ROBINS-I tool for non-RCT studies, there was very serious risk of bias related to confounding variables, classification of interventions, deviations from the intended interventions, missing data and the measurement of outcomes across the two studies. We downgraded by 2.
r There was inconsistency in the tools used to measure satisfaction but both studies showed a positive effect. We downgraded by 0.5.
s The total number of participants is far less than the optimal 800 participants (n=113). We downgraded by 2.
t Based on the risk-of bias-tool for randomized trials (RoB 2), there was serious risk of bias related to the randomization process, deviations from the intended interventions, and the selection of reported results across the five studies. We downgraded by 1.5
u There were inconsistent results among the five studies. We downgraded by 1.5.
v The number of events is greater than 300. We did not downgrade.
w Based on the ROBINS-I tool for non-RCT studies, there was serious risk of bias related to confounding variables and deviations from the intended interventions. We downgraded by 1.5.
x There was likely around 300 readmissions but the total number of participants was less than the optimal 800 participants (n= 439 participants). We downgraded by 0.5.
y Based on the ROBINS-I tool for non-RCT studies, there was serious risk of bias related to confounding variables, deviations from the intended interventions and missing data. We downgraded by 1.5.
z The total number of participants is less than the optimal 800 participants (n=586). We downgraded by 1.

References

1. Rose L, Istanboulian L, Carriere L, et al. Program of integrated care for patients with chronic obstructive pulmonary disease and multiple comorbidities (PIC COPD+): a randomised controlled trial. *Eur Respir J* 2018; 51: 1701567.

2. Reeves M, Fritz M, Woodward A, et al. Michigan stroke transitions trial: a clinical trial to improve stroke transitions. *Circ Cardiovasc Qual Outcomes* 2019; 12: e005493.

3. Ng A, Wong F. Effects of a home-based palliative heart failure program on quality of life, symptom burden, satisfaction and caregiver burden: a randomized controlled trial. *Journal of pain and symptom management* 2018; 55: 1–11.

4. Kidd SA, Virdee G, Mihalakakos G, et al. The welcome basket revisited: testing the feasibility of a brief peer support intervention to facilitate transition from hospital to community. *Psychiatr Rehabil J* 2016; 39: 335–342.

5. Balaban R, Zhang F, Vialle-Valentin C, et al. Impact of a patient navigator program on hospital-based and outpatient utilization over 180 days in a safety-net health system. *J Gen Intern Med* 2017; 32: 981.

6. Carter J, Hassan S, Walton A, et al. Effect of community health workers on 30-day hospital readmissions in an accountable care organization population: a randomized clinical trial. *JAMA Netw Open* 2021; 4: e2110936–e2110936.

7. Mitchell SE, Reichert M, Martin Howard J, et al. Reducing readmission of hospitalized paatients with depressive symptoms: a randomized trial. *Annals of Family Medicine* 2022; 20: 246–254.

8. Taylor SPM, Murphy SD, Rios AN, et al. Effect of a multicomponent sepsis transition and recovery program on mortality and readmissions after sepsis: the improving morbidity during post-acute care transitions for sepsis randomized clinical trial. *Critical Care Medicine* 2022; 50: 469–479.

9. Seaberg D, Elseroad S, Dumas M, et al. Patient navigation for patients frequently visiting the emergency department: a randomized controlled trial. *Acad Emerg Med* 2017; 24: 1327–1333.

10. LaBedz SL, Prieto-Centurion V, Mutso A, et al. Pragmatic clinical trial to improve patient experience among adults during transitions from hospital to home: the PArTNER study. *JGIM: Journal of General Internal Medicine* 2022; 37: 4103–4111.

11. Pang RK, Srikanth V, Snowdon DA, et al. Targeted care navigation to reduce hospital readmissions in 'at-risk' patients. *Intern Med J*. Epub ahead of print 2023. DOI: 10.1111/imj.15634.

12. Johnson S, Lamb D, Marston L, et al. Peer-supported self-management for people discharged from a mental health crisis team: a randomised controlled trial. *Lancet* 2018; 392: 409.

13. Samuels EA, Kelley L, Pham T, et al. 'I wanted to participate in my own care': evaluation of a patient navigation program. *West J Emerg Med* 2021; 22: 417–426.

14. Scanlan JN, Hancock N, Honey A. Evaluation of a peer-delivered, transitional and post-discharge support program following psychiatric hospitalisation. *BMC Psychiatry* 2017; 17: 307.

15. Coller R, Klitzner T, Lerner C, et al. Complex care hospital use and postdischarge coaching: a randomized controlled trial. *Pediatrics*; 142.

16. Thompson MP, Podila PSB, Clay C, et al. Community navigators reduce hospital utilization in super-utilizers. *Am J Manag Care* 2018; 24: 70–76.

17. Xiang X, Zuverink A, Rosenberg W, et al. Social Work-based transitional care intervention for super utilizers of medical care: a retrospective analysis of the bridge model for super utilizers. *Soc Work Health Care* 2019; 58: 126–141.
