# Supplementary material for: Navigation Support during Transitions in Care for Persons with Complex Care Needs: A Systematic Review
Source: Healthcare (Basel). 2024 Sep 10;12(18):1814. doi: 10.3390/healthcare12181814 (PMC11431248; doi:10.3390/healthcare12181814)
Supplement: Supplementary file 1 [file healthcare-12-01814-s001.zip › File S3_Intervention duration.pdf]

File S3: Patient satisfaction and quality of life listed in order of intervention duration

Patient satisfaction listed in order of intervention duration

**Approximate duration of intervention**	Studies	Outcome measurement	Summary of findings: patient satisfaction
**6-8 weeks**	Scanlan et al., 2017	Questionnaire completed at program conclusion to measure satisfaction with intervention	The program appeared to be valuable for participants who received the intervention.
**3-4 months**	Johnson et al., 2018	Client Satisfaction Questionnaire completed at 4 months to measure respondents' satisfaction with mental health services	At program conclusion, satisfaction was greater in the intervention group than the control group in both studies.
	Ng & Wong, 2018	Patient satisfaction questionnaire completed at 4 weeks and 12 weeks to measure satisfaction with the post discharge care	
**1 year**	Samuels et al., 2021	Program satisfaction was assessed at 1 year using telephone surveys	Two studies showed no important differences in patient satisfaction between the intervention and control groups (Rose et al., 2018; Seaberg et al., 2017). In Samuel et al.'s study, participants reported high satisfaction with navigation support after the program was complete.
	Rose et al., 2018	Client Satisfaction Questionnaire-8 completed at baseline, 3, 6 and 12 months	
	Seaberg et al., 2017	Satisfaction with services was assessed at 2 weeks and 12 months using a telephone survey	

Patient quality of life listed in order of intervention duration

**Approximate duration of intervention**	Studies	Outcome measurement	Summary of findings: Patient quality of life
**1 month**	Kidd et al., 2016	Satisfaction With Life Scale was completed pre and post intervention	Post intervention, there was an improvement in self-reported QOL in the domains of 'living situation' with a large effect size and 'social relationships' with a low-medium effect size. There were no differences in the QOL domains of 'self and present life' or 'work'.
**2 months**	Reeves et al., 2019	Patient-Reported Outcomes Measurement Information System Global-10, a generic QOL questionnaire composed of physical and mental health subscales was completed at 7 days and 90 days of returning home	Post intervention, there were no important differences in physical or mental health QOL between the intervention and control group.
**3 months**	Ng & Wong, 2018	McGill Quality of Life Questionnaire-Hong Kong and the Chronic Heart Failure Questionnaire-Chinese were completed before discharge and at 4 weeks and 12 weeks after discharge	Post intervention, QOL was greater in the intervention group than the control group over time when MQOL-HK scores were compared.<br><br>At 4 weeks, CHQ-C scores were greater in the intervention group compared to the control group. These differences were not seen at 12 weeks.
**1 year**	Rose et al., 2018	Generic and disease-specific quality of life questionnaires were completed at baseline, 3, 6 and 12 months	Post intervention, there were no important differences in generic or disease specific QOL between the intervention and control group.

Abbreviations: Quality of life (QOL)
